# MoO_3_/ZnMoO_4_ Composite Obtained in a One-Pot Reaction and Applied in the Production of Biodiesel from Acid Feedstocks

**DOI:** 10.1021/acsomega.6c05074

**Published:** 2026-09-22

**Authors:** Heritier Kabamba Subissay, Amanda Soares de Sousa, Ana Cristina F. M. Costa, Ieda Maria Garcia dos Santos

**Affiliations:** † Núcleo de Pesquisa e Extensão − Laboratório de Combustíveis e Materiais, 28097 Universidade Federal da Paraíba, Joao Pessoa, Paraíba CEP 58051-900, Brazil; ‡ Laboratório de Síntese dos Materiais Cerâmicos (LabSMaC), Universidade Federal de Campina Grande, Campina Grande, Paraíba 58429-900, Brazil

## Abstract

Despite being widely recognized within the scientific community, the synthesis of biodiesel from acidic feedstocks remains challenging. In this work, a heterogeneous catalyst comprising molybdenum trioxide and zinc molybdate (MoO_3_/ZnMoO_4_) was synthesized via the modified Pechini method and characterized to assess its structural, morphological, and chemical properties. The catalytic performance of MoO_3_/ZnMoO_4_ in esterification, transesterification, and simultaneous esterification-transesterification reactions (SET) at different temperatures and times of reaction was evaluated. α-MoO_3_ and ZnMoO_4_, with high crystallinity, were obtained, exhibiting Lewis acid sites and a negatively charged surface across all pH values. Heterogeneous morphology identified by SEM/EDS, including ribbon-like and prismatic particles characteristic of α-MoO_3_, alongside irregular micrometric particles attributed to the ZnMoO_4_ phase. The composite exhibited strong catalytic activity in transesterification, achieving conversion of 76.61% at 160 °C/120 min and 82.84% at 200 °C/120 min—both exceeding values reported for pristine ZnMoO_4_. In esterification, conversions ranged around 96% and 97%, with limited dependence on temperature and reaction time, suggesting that milder conditions are sufficient for high efficiency. In the SET reaction, conversions of about 90% were obtained using oil acidified with 10% or 20% oleic acid, and 96.22% conversion was achieved with crude palm oil, demonstrating strong tolerance toward acidic feedstocks. Moreover, the material kept its catalytic activity after 4 reuse tests. The superior performance of the composite is primarily attributed to the synergistic interaction between ZnMoO_4_ and MoO_3_.

## Graphical abstract

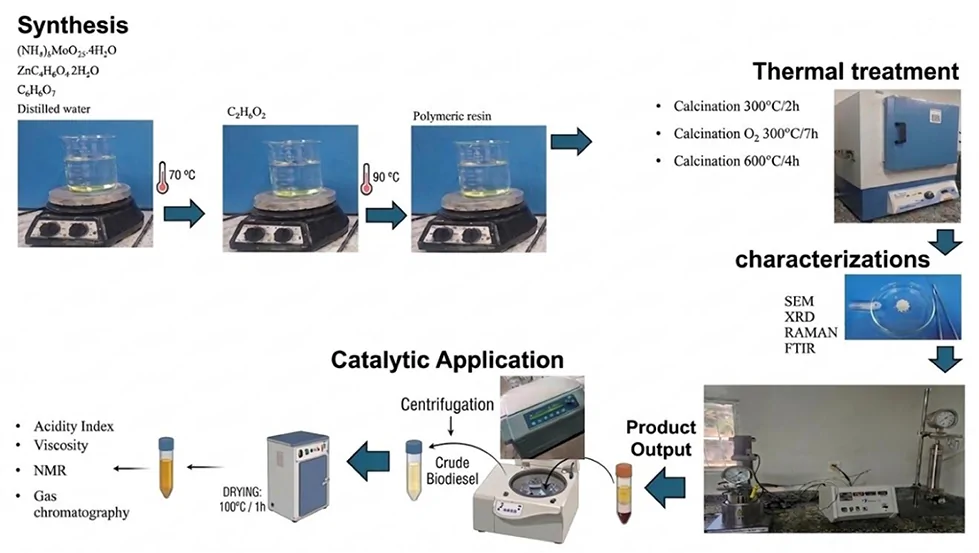

## 1 Introduction

The intensification of environmental impacts associated with fossil fuel consumption, particularly greenhouse gas emissions, has driven a global transition toward renewable-energy sources. In this context, biodiesel has gained prominence as a renewable, biodegradable, and low-toxicity biofuel that significantly reduces emissions compared to fossil diesel and can be used neat or blended in conventional engines without major modifications.^1^

Biodiesel is produced through the transesterification of triglycerides or the esterification of fatty acids with short-chain alcohols; however, homogeneous catalysts present significant drawbacks, including soap formation, high water consumption, and complex separation processes.^2^ Heterogeneous catalysts have therefore emerged as a more attractive alternative, offering greater stability, selectivity, reusability, and reduced waste generation, thereby making the process more sustainable.^3–5^ Their use becomes even more attractive when acidic feedstocks—such as residual oils—are employed, as these catalysts can promote the simultaneous esterification of free fatty acids and transesterification of triglycerides.^6^

Among the materials investigated for heterogeneous catalysis in biodiesel synthesis, molybdenum trioxide (α-MoO_3_) has attracted considerable attention owing to its Brønsted–Lowry and Lewis acid sites, high thermal stability, and crystalline structures favorable for the activation of lipid molecules.^7–9^ To further enhance its catalytic performance, several strategies have been explored, including composite formation,^1,10^ crystalline phase control,^11^ structural modifications induced by calcination temperature,^12^ and dispersion onto various supports.^13–18^

Beyond α-MoO_3_, transition-metal molybdates have also been investigated in biodiesel-related reactions. For instance, AlKahlaway et al.^19^ synthesized Fe_2_(MoO_4_)_3_ via the sol–gel method and applied it in the esterification of oleic acid with ethanol, reaching 92.5% conversion at 70 °C after 5 h. Silva Junior et al.^20^ synthesized Cu_3_(MoO_4_)_2_(OH)_2_ by a sonochemical route and evaluated it in the esterification of oleic acid with methanol, reaching 98.38% conversion at 140 °C after 5 h, with good stability over five reuse cycles. Additionally, our research group previously synthesized α-ZnMoO_4_ via the modified Pechini method and assessed its catalytic activity in transesterification, esterification, and simultaneous esterification–transesterification (SET) reactions.^3^ Although the pristine material did not achieve high conversion in transesterification alone, a synergistic effect was observed when acidified soybean oil was used: the esterification reaction appeared to induce the formation of Brønsted–Lowry acid sites, which in turn favored transesterification.

In the present work, a composite of these two materials—ZnMoO_4_ and MoO_3_—was synthesized via the modified Pechini method in a one-pot reaction and evaluated as a catalyst for biodiesel production, with a focus on its physicochemical, structural, and catalytic properties.

## 2 Materials and Methods

### 2.1 Synthesis and Characterization of MoO_3_/ZnMoO_4_

The MoO_3_/ZnMoO_4_ composite was synthesized via the modified Pechini method, with simultaneous crystallization of both phases. The precursors used were zinc acetate (ZnC_4_H_6_O_4_·2H_2_O, Vetec, 98%); ammonium molybdate (NH_4_)_6_Mo_7_O_24_·4H_2_O, (Moderna, 83%); citric acid C_6_H_8_O_7_ (Vetec, 99.5%); and ethylene glycol HOCH_2_CH_2_OH (Vetec, 99.0%).

The precursor solution was prepared in a 500 mL beaker containing 250 mL of distilled water, maintained at 70 °C under constant stirring. Citric acid (9.415 g), ammonium molybdate (1.9220 g), and zinc acetate (1.1949 g) were added sequentially at a metal cation-to-citric acid molar ratio of 1:3. After complete dissolution, the temperature was raised to 90 °C, and 5.64 mL of ethylene glycol was added, maintaining a citric acid-to-ethylene glycol mass ratio of 60:40. This promoted polymerization of the organic matrix and resin formation, after which the material was subjected to heat treatment (Figure 1). The synthesized material was characterized by X-ray diffraction (XRD), infrared spectroscopy (IR), Raman spectroscopy, zeta potential measurements, BET surface area analysis, and scanning electron microscopy (SEM).

**Figure 1:**
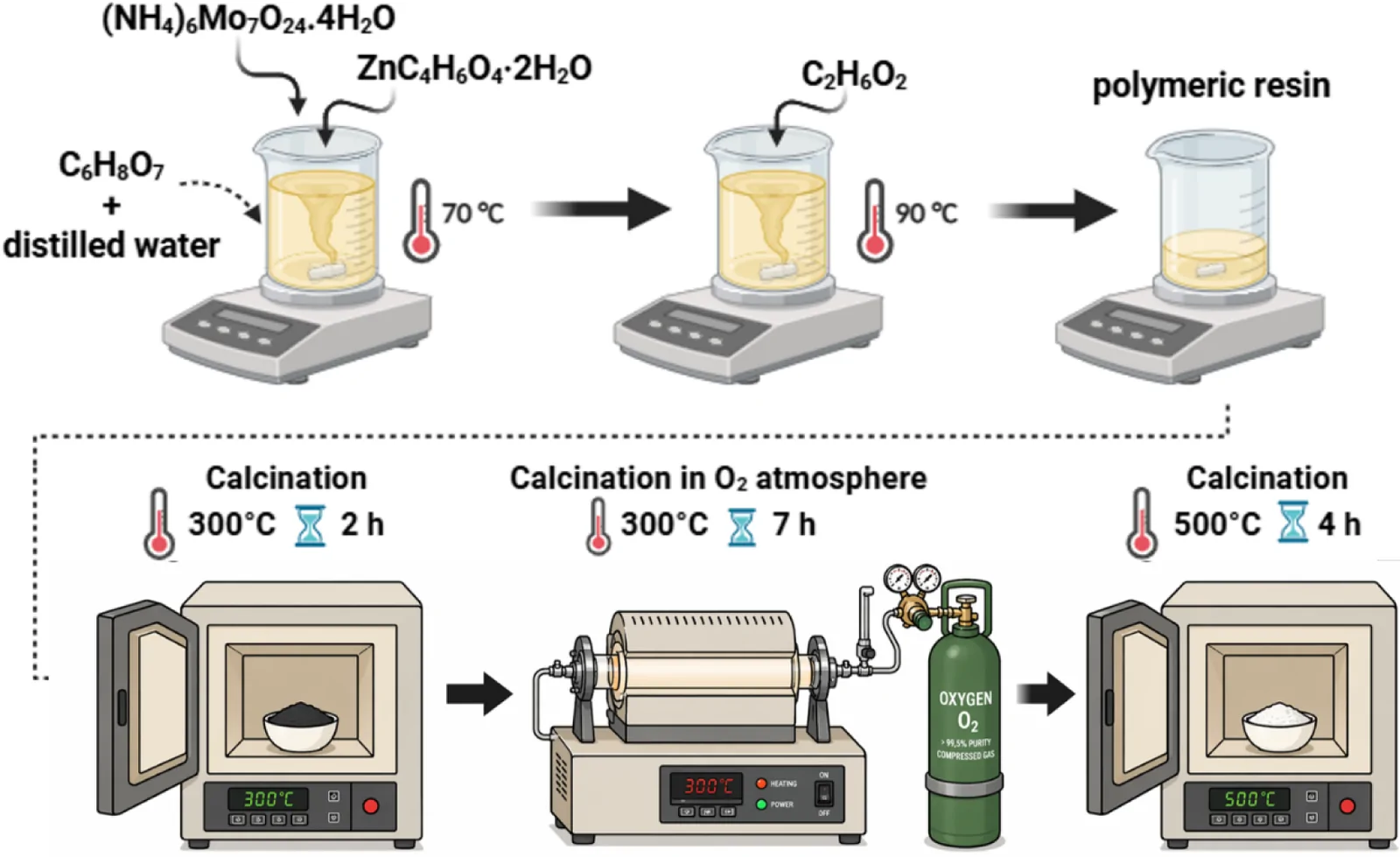
Schematic diagram of the synthesis of the MoO_3_/ZnMoO_4_ composite by the modified Pechini method. Figure created using BioRender.com and complemented with AI-generated elements by ChatGPT, followed by manual editing.

The structural characterization of the samples was performed via X-ray diffraction (XRD) using a Shimadzu Lab X/XRD-6000 diffractometer. The equipment operated using Cu–Kα radiation at a power of 2 kVA, with a voltage of 30 kV and a current of 30 mA. Data collection was carried out through 2θ scans ranging from 10° to 80° with a step size of 0.02° and a scanning speed of 2° min^–1^. Structural refinement was performed using the RIETVELD method and the Maud software.

Infrared spectrum (FTIR) was acquired in transmittance mode within the 400–4000 cm^–1^ spectral range by a Shimadzu IRPrestige-21 spectrophotometer. Samples were prepared using the KBr pellet technique. Raman spectrum was recorded at room temperature over a spectral range of 100 to 1000 cm^–1^, using a RENISHAW InVia spectrometer equipped with an argon laser operating at a wavelength of 514 nm and a power of 200 mW. The analyses were conducted using a 50× objective lens.

Textural characterization was performed by N_2_/He adsorption using an Autosorb surface area analyzer (Quantachrome Instruments), and the specific surface area was determined by the Brunauer–Emmett–Teller (BET) method. The morphological characterization of the MoO_3_/ZnMoO_4_ catalyst was performed by scanning electron microscopy (SEM) using a TESCAN VEGA4 LMS microscope equipped with an Essence energy-dispersive spectroscopy (EDS) system. The samples were dispersed on double-sided carbon tape, fixed to an aluminum support, and coated with a thin layer of gold to ensure the electrical conductivity during the analysis.

The characterization of the acid sites was carried out by FT-IR using pyridine (Py) as a probe molecule. Initially, the sample was treated at 100 °C for 1 h under N_2_ flow, and subsequently placed in a sealed container containing liquid pyridine, without direct contact with the sample, under vacuum. The system was maintained under these conditions for 72 h to allow the pyridine vapor to interact with the acidic sites of the solid.^3^ Subsequently, the samples were treated at 100, 200, 300, and 400 °C to evaluate the desorption of the adsorbed pyridine. The spectra were recorded on a Shimadzu IR Prestige-21 spectrometer in the 1400–1700 cm^–1^ region. The amount of Lewis acid sites was estimated from the integration of the band at 1448 cm^–1^ using the equation described by del Rey-Perez-Caballero and Poncelet.^21^

### 2.2 Catalytic Tests

The catalytic activity of MoO_3_/ZnMoO_4_ was evaluated in the transesterification of commercially refined soybean oil and the esterification of oleic acid (p.a., Synth) using methanol (99.8%, Synth) as the acyl acceptor. Reactions were conducted in a Parr autoclave reactor (model 4561, 300 mL) operating in batch mode, with a methanol-to-substrate molar ratio of 12:1 and a substrate charge of 20 g per experiment. The reactions were performed at 160 and 200 °C for 30, 60, and 120 min, using a catalyst loading of 3 wt % relative to the organic substrate and constant stirring at 500 rpm. After each reaction, the products were characterized by kinematic viscosity and acid value measurements, while chemical composition and conversion were determined by ^1^H and ^13^C Nuclear Magnetic Resonance (NMR) spectroscopy and gas chromatography (GC).

The viscosity of the product was measured using a Julabo V18 model viscometer and a Cannon-Fenske size 150 glass capillary viscometer, operating at a temperature of 40 °C.

For acidity analysis, a potassium hydroxide (KOH) solution was previously prepared and standardized using potassium biphthalate (KHC_8_H_4_O_4_) as a primary standard. For the analysis of the reaction products, approximately 1.0 g of the sample was weighed into an Erlenmeyer flask, dissolved in 10 mL of toluene, and titrated with the standardized KOH solution, in the presence of phenolphthalein, until the indicator changed color. The tests were performed in duplicate. Reaction products were characterized by the acid value, according to the AOCS. The acid value, expressed in mg KOH g^–1^, was calculated according to eq 1.

1 $$A.I.=\frac{\left(V-V_{B}\right)\times F\times M\times 56.1}{m}$$

where V is the volume of titrant used by the sample, V_B_ is the volume used in the blank, F is the correction factor of the KOH solution, M is the molarity of the base, and m is the sample mass (g).

The conversion of oleic acid to methyl esters by acidity measurements (C_AI_) was calculated from the initial (AI*_i_*) and final (AI*_f_*) values of acid value, according to eq 2.^22^

2 $$C_{AI}\left(\%\right)=\frac{{AI}_{i}-{AI}_{f}}{{AI}_{i}}\times 100$$

^1^H and ^13^C Nuclear Magnetic Resonance (NMR) spectra were acquired using a Bruker Ascend 400 spectrometer, operating at 400 MHz for ^1^H and 100 MHz for ^13^C. For each analysis, approximately 30 mg of the sample was dissolved in 0.6 mL of deuterated chloroform (CDCl_3_). The spectra were processed using MestReNova software, with chemical shifts expressed in ppm, and the ^13^C spectra were acquired using the APT technique.

The conversion of oil to biodiesel (%C_ME_) was determined from ^1^H NMR spectral integration following the methodology proposed by Ruschel et al.,^23^ and calculated using eq 3,^24^ based on the ratio between the integrated areas of the signals corresponding to the hydrogens of the methoxyl protons of the methyl ester (δ 3.6 ppm) and the methylene protons adjacent to the carbonyl (α-CH_2_, δ 2.3 ppm):

3 %CME=100×(IME3Iα‐CH22)

where %C_ME_ corresponds to the percent conversion to methyl esters, I_ME_ is the integrated area of the COOCH_3_ signal, and $I_{CH_{2}}$ is the integrated area of the α-methylene signal.

The products of the transesterification reaction were also characterized by gas chromatography to quantify the conversion to esters, using a Varian 450c gas chromatograph equipped with a flame ionization detector (FID) and a Varian Ultimetal Select Biodiesel Glycerides + RG capillary column (15 m × 0.32 mm × 0.45 μm).

## 3 Results and Discussion

### 3.1 Characterization of the MoO_3_/ZnMoO_4_Characterization of the MoO/ZnMoO Composite

The XRD pattern of the MoO_3_/ZnMoO_4_ sample, synthesized by the modified Pechini method and calcined at 600 °C, displays characteristic peaks of both MoO_3_ and ZnMoO_4_ crystalline phases (Figure 2). Phase identification was performed using ICDD files 01-070-1003 (MoO_3_) and 01-070-5387 (ZnMoO_4_), confirming the formation of the expected two-phase composite. The XRD pattern exhibits high crystallinity, as evidenced by narrow, intense peaks.

**Figure 2:**
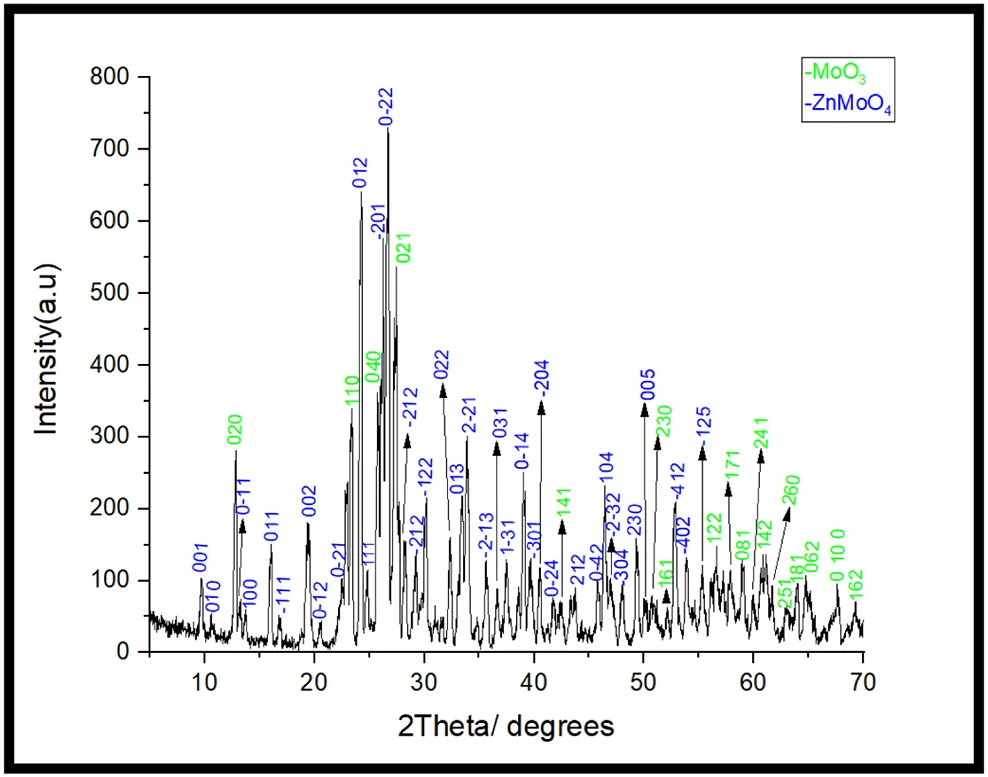
Indexed XRD pattern of the MoO_3_/ZnMoO_4_ composite.

Rietveld refinement yielded an excellent fit (R_wp_, R_b_, and GOF < 5%), with strong agreement between experimental and calculated profiles (Figure SI.1), ruling out the presence of secondary phases or impurities. The results are summarized in Table 1. Phase quantification indicates a predominance of ZnMoO_4_ (68.64%) over that of MoO_3_ (31.35%), consistent with the relative intensities of the diffraction peaks. Crystallite size estimates confirm the nanocrystalline character of both phases, with ZnMoO_4_ exhibiting a smaller average crystallite size and lower microstrain, indicative of a better crystal ordering.

**Table 1:** Rietveld Refinement Results for the MoO_3_/ZnMoO_4_ Composite^a^

	ZnMoO_4_	MoO_3_
Lattice parameters (Å)	a = 6.96	a = 3.963
	b = 8.36	b = 13.885
	c = 9.69	c = 3.695
Crystalline system	Triclinic	Monoclinic
Space group	P-1	C2/m
Unit cell volume (A^3^)	519.81	202.45
Phase percentage (%)	68.64	31.35
Crystallite size (nm)	70	220
Microstrain	0.00148	0.00289
GOF	2.26	1.41
R_wp_ (%)	3.40	1.72
R_b_ (%)	3.47	1.27
R_exp_	2.19	1.92

a Based on the ICDD crystallographic files 01-070-5387 and 01-070-100.

FTIR and Raman spectra (Figure 3) confirm the formation of the MoO_3_/ZnMoO_4_ composite, with vibrational bands assigned to both phases in agreement with the XRD results.

**Figure 3:**
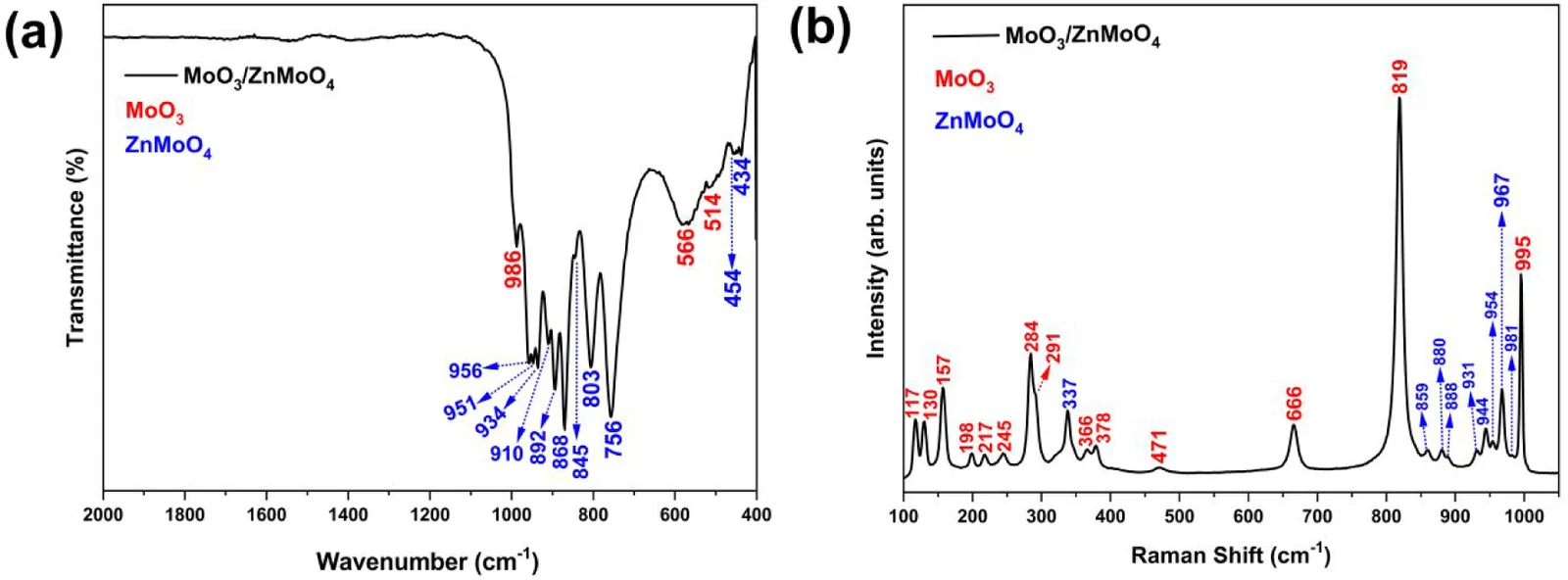
Spectroscopic characterization of the MoO_3_/ZnMoO_4_ composite: (a) FTIR spectrum; (b) Raman spectrum.

The infrared spectral profile (Figure 3a) more closely resembles that of ZnMoO_4_ than MoO_3_, corroborating the structural analysis. The predominance of ZnMoO_4_ is particularly evident in the 750–960 cm^–1^ region, associated with Mo–O stretching vibrations in tetrahedral sites and Zn–O stretching in octahedral sites, characteristic of the ZnMoO_4_ crystal structure. Bands attributed to MoO_3_ appear with lower intensity, most notably at 986 cm^–1^ and in the 500–600 cm^–1^ range, associated with terminal Mo=O stretching and lattice deformation modes of this phase. Their presence confirms the coexistence of MoO_3_ in the composite, albeit as the minority phase, consistent with XRD quantification. Band assignments are provided in Table SI.1.

Comparison with literature spectra reveals a pronounced shift in the Mo(3)–O and δ­(O–Mo–O) bands of MoO_3_, with a smaller shift observed for the terminal Mo=O bond. For ZnMoO_4_, the band at 434 cm^–1^ shows reduced intensity, while the band at approximately 454 cm^–1^—previously observed only as a shoulder in pure ZnMoO_4_ ^3^—becomes clearly resolved. Analogous behavior is observed for the bands in the 934–956 cm^–1^ range, along with minor additional shifts near 756 and 803 cm^–1^.

The Raman spectrum (Figure 3b) confirms the high short-range order of the composite, with MoO_3_ peaks dominating the full spectral range, while ZnMoO_4_ peaks appear between 860 and 970 cm^–1^ at lower intensity. As reported by Kassem,^25^ the low-symmetry and distorted structural units of α-ZnMoO_4_ give rise to distinct Zn–O–Mo bond environments, producing a peak at 970 cm^–1^—the most intense band assigned to ZnMoO_4_. Vibrational mode assignments are listed in Table SI.2. Frequency shifts for MoO_3_ remain below 5 cm^–1^, whereas the MoO_4_ tetrahedral stretching bands of ZnMoO_4_ show greater sensitivity to interactions within the composite, suggesting higher structural flexibility for this phase.

Together, FTIR and Raman spectroscopy provide complementary and consistent evidence for the interaction between the two phases. The modifications observed in the characteristic vibrational modes of both crystalline structures are attributed to interface effects, which disturb the local symmetry and vibrational coupling of metal–oxygen groups as a result of their simultaneous crystallization.

The BET surface area of the catalyst was 1.8 m^2^ g^–1^, slightly higher than values reported in the literature. For comparison, Colaço et al.^3^ obtained 0.37 m^2^ g^–1^ for ZnMoO_4_ synthesized by the same method and calcined at 700 °C, while our research group obtained 0.50 m^2^ g^–1^ was for pristine MoO_3_.^26^ Using the phase fractions from Rietveld refinement and the theoretical densities of both phases, the density of the composite was estimated. Combined with the BET surface area, this yielded a BET particle diameter (D_BET_) of 0.75 μm—more than 10 times the crystallite size of ZnMoO_4_ and approximately 3.4 times that of MoO_3_—suggesting sintering between particles during synthesis.

Although surface area is generally correlated with catalytic activity, as larger areas increase the number of accessible active sites, a low surface area can be compensated by the presence of highly active surface sites, as previously demonstrated by our research group for MoO_3_ in soybean oil transesterification.^7,26^

SEM micrographs and the EDS mapping (Figure 4) reveal distinct morphological features for each phase in the composite. MoO_3_ exhibits prismatic and ribbon-like particles with well-defined micrometric dimensions in the 2–5 μm range, consistent with its high crystallinity and anisotropic growth, as reported by Medeiros et al.^7^ The absence of Zn in the prismatic particle confirms that it is solely composed of MoO_3_. ZnMoO_4_, in contrast, presents a cluster-like morphology indicative of less oriented crystal growth, attributed to the influence of Zn^2+^ ions on nucleation and crystallization dynamics, which favors the formation of micrometric and submicrometric irregular particles.^3^ A superposition of Mo and Zn is clearly observed in the EDS map and confirms that these irregular particles are composed of ZnMoO_4_. Oxygen is distributed throughout the image because material was deposited on the Si substrate, which has a SiO_2_ film on its surface. This morphological architecture is fully consistent with the findings of Medeiros et al.^7^ for MoO_3_ and Colaço et al.^3^ for ZnMoO_4_, confirming that the composite retains the fundamental morphological characteristics of each phase.

**Figure 4:**
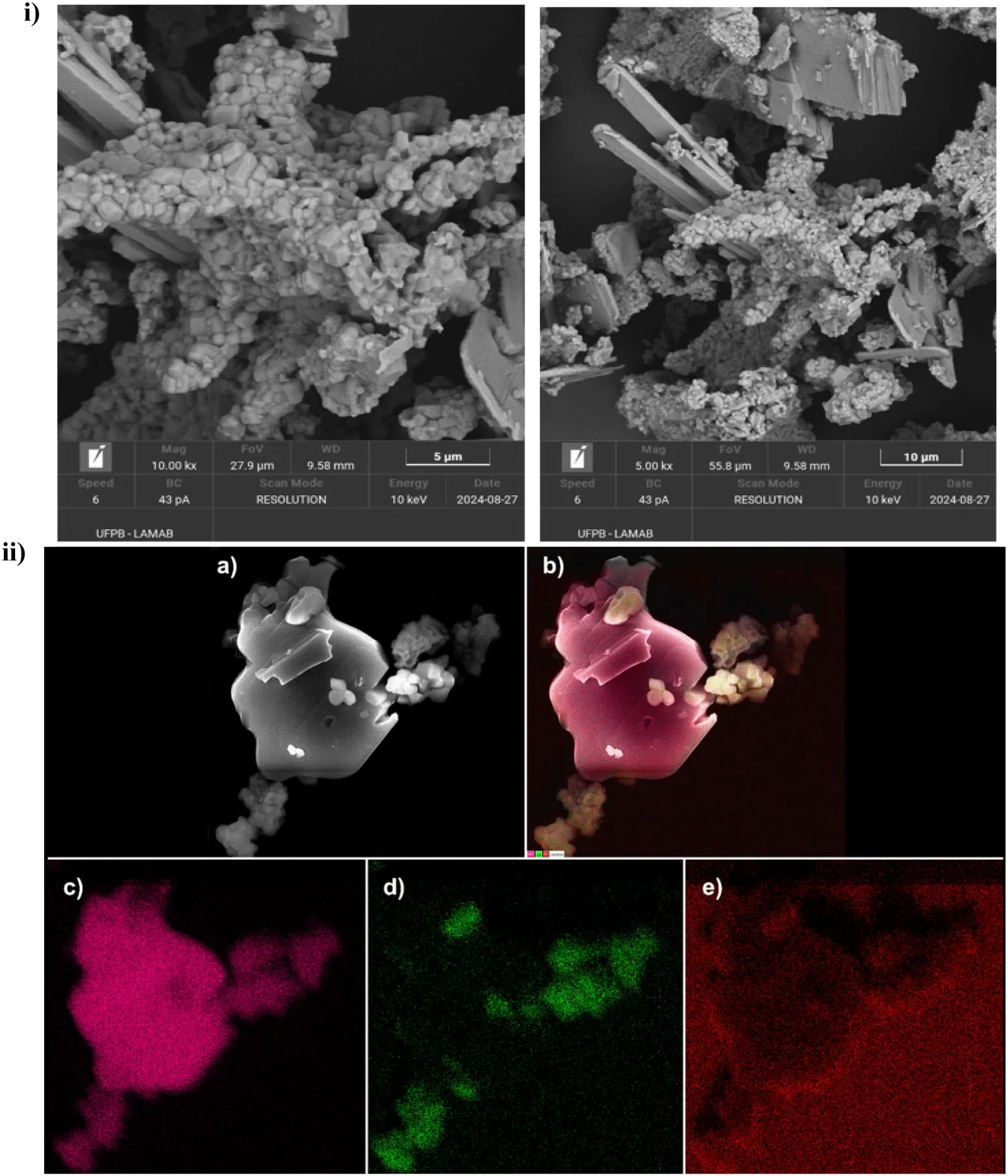
(i) SEM micrographs of the MoO_3_/ZnMoO_4_ composite; ii) EDS mapping showing the distribution of the elements in the different phases of the composite: a. SEM micrograph; b. phase composition; c. Mo map; d. Zn map; e. O map.

Pyridine adsorption FTIR spectroscopy was used to identify and quantify the acid sites on the composite surface. Spectra recorded at 25 °C and after stepwise desorption at 100, 200, 300, and 400 °C are shown in Figure 5. The spectrum at 25 °C displays five bands at approximately 1448, 1486, 1572, 1606, and 1640 cm^–1^. According to Zaki et al.,^27^ the ν_19b_ (1448 cm^–1^) and ν_8a_ (1603 cm^–1^) stretching modes are assigned to pyridine coordinated to Lewis acid sites, while the band at 1486 cm^–1^ is attributed to pyridine adsorbed on both Brønsted–Lowry and Lewis sites.

**Figure 5:**
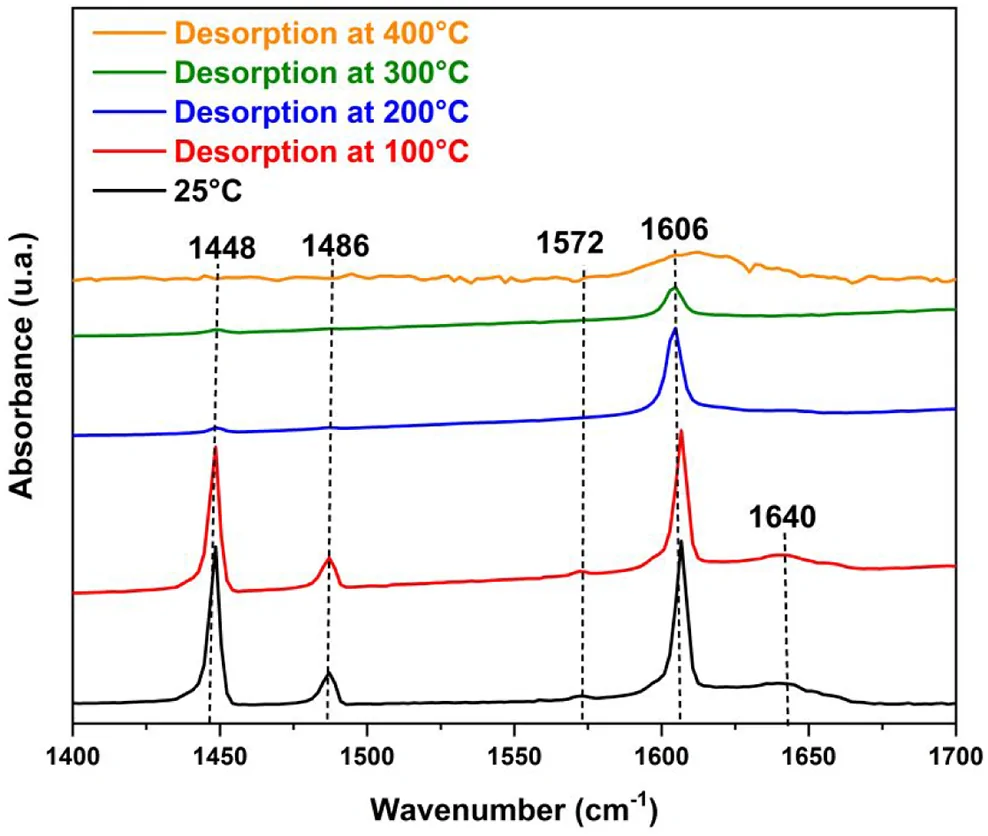
FTIR spectra of pyridine adsorbed on MoO_3_/ZnMoO_4_ at 25 °C and after desorption at 100, 200, 300, and 400 °C.

The band in the 1500–1540 cm^–1^ range (ν_19b_), characteristic of pyridinium ion formation and indicative of the presence of Brønsted–Lowry acid sites, was absent. The weak peak at 1640 cm^–1^ is therefore most likely assigned to pyridine coordinated to stronger Lewis acid sites.^27^ With increasing desorption temperature, all bands progressively decrease in intensity; after treatment at 400 °C, only a residual band at 1606 cm^–1^ remains.

The number of Lewis acid sites quantified by pyridine FTIR is presented in Table 2. Due to potential band overlap in the 1445–1460 cm^–1^ and 1600–1633 cm^–1^ regions from physisorbed (PPy) or hydrogen-bonded pyridine (HPy), acid site quantification was based on spectra acquired after desorption at 200 °C, at which weakly adsorbed species are removed.^3,28^ The number of sites was divided by the BET surface area in order to obtain the surface density of acid sites.

**Table 2:** Lewis Acid Site Content of the MoO_3_/ZnMoO_4_ Composite Determined by the Pyridine-Probe Method^a^

Temperature	q_L_ (μmol g^–1^)	Number of sites (μmol g^–1^)	Density of sites (μmol m^–2^)	Assignment
25 °C	66.11	62.81	34.9	HPy
100 °C	54.80			
200 °C	3.30	1.02	0.57	LPy (weak)
300 °C	2.28	2.28	1.27	LPy (moderated)
400 °C	0.0	0.0	0.0	LPy (strong)

a q_L_—Number of Lewis sites evaluated by integrating the absorption band area at 1448 cm^–1^; HPy—hydrogen-bonded pyridine; LPy—pyridine adsorbed on Lewis acid sites.

Published data on MoO_3_-based materials generally report the coexistence of Brønsted–Lowry and Lewis acid sites, with Lewis sites predominating.^11,29,30^ For ZnMoO_4_, only one study has been reported to date,^3^ and studies on transition-metal molybdates remain scarce.^31^ Colaço et al.^3^ found that pure ZnMoO_4_ exhibited exclusively Lewis acid sites, evidenced by bands at 1448, 1486, and 1606 cm^–1^, with a total number of acid sites (q_T_) of approximately 181 μmol g^–1^, mostly of weak-to-moderate strength. Sousa et al.^26^ also synthesized α-MoO_3_ by the modified-Pechini method and observed Lewis and Brønsted acid sites on the material surface, with q_T_ = 55 μmol g^–1^. In the MoO_3_/ZnMoO_4_ composite, the number of weak and moderate Lewis sites is lower (3.30 μmol g^–1^) than in both pure phases. This value is even smaller, considering the surface density of acid sites, since Colaço and Sousa obtained smaller surface areas than the present work. The main difference between the pristine ZnMoO_4_ and the composite obtained in the present work may be related to new bands at 1572 and 1640 cm^–1^, suggesting the formation of distinct pyridine coordination environments on the composite surface, likely arising from interface effects associated with the presence of MoO_3_, as corroborated by both FTIR and Raman spectroscopy (Figure 3).

### 3.2 Catalytic Tests

After the catalytic tests, the reaction products were characterized by kinematic viscosity, acid value, GC, and ^1^H and ^13^C NMR. Viscosity data are provided in the SI; the remaining characterization techniques are discussed in the following sections.

#### 3.2.1 Transesterification Reaction

The initial acid value (AI) of soybean oil was 2.0 mg KOH g^–1^. After reaction, product acid values ranged from 0.98 to 4.7 mg KOH g^–1^, with an increase in free fatty acid (FFA) content at longer reaction times.

The ^1^H NMR spectra (Figure 6) confirm the conversion of triglycerides into methyl esters (MEs) evidenced by the intense signal at 3.65–3.66 ppm attributed to the methoxyl group (−OCH_3_).^23,32^ The signal intensity increases with reaction time, indicating progressive conversion of triacylglycerols. The signals in the 4.1–4.3 ppm region, characteristic of the glyceride backbone in crude soybean oil, diminish progressively after transesterification, reflecting the formation of ME and glycerol.^33^

**Figure 6:**
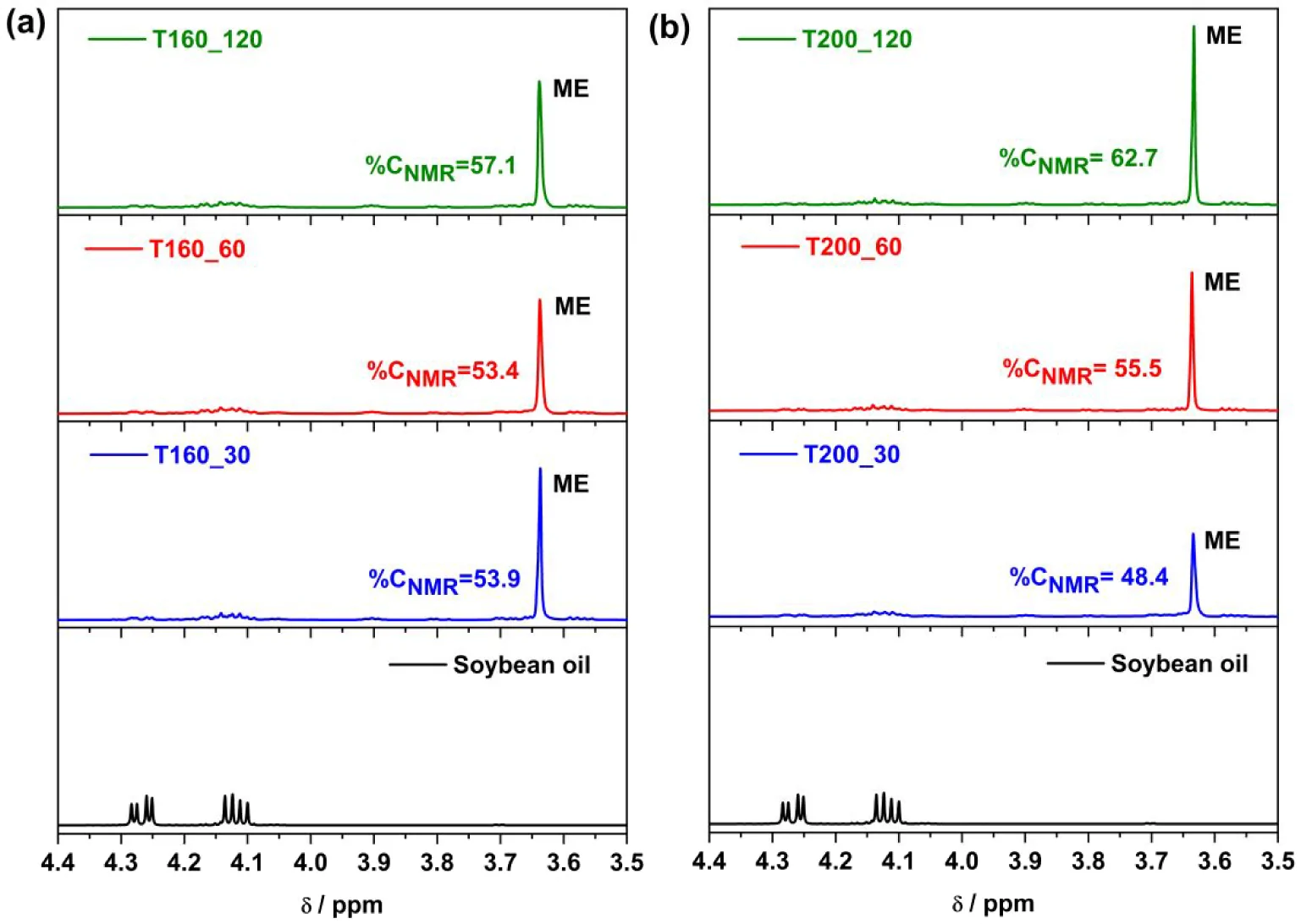
Expanded ^1^H NMR spectra (4.4–3.5 ppm) of soybean oil and transesterification products at (a) 160 °C and (b) 200 °C.

The highest conversion was observed for sample T200_120 (62.7%), followed by T160_120 (57.1%), confirming that a higher temperature and longer reaction time favor conversion. The nonlinear increase in conversion suggests the system approaches chemical equilibrium near 120 min, a behavior typical of heterogeneous catalytic systems.^33^ Residual signals from the glyceride portion of the triacylglycerols are still observed.

The ^13^C NMR spectra (Figure SI.3) corroborate the partial nature of the transesterification. The characteristic signal of the methyl ester carbonyl (δ_C_ = 51.4 ppm) confirms ME formation from soybean oil triglycerides.^34^ However, persistence signals in the 60–70 ppm region, attributed to residual triglycerides and mono- and diglyceride intermediates, indicate that the reaction did not reach completion. The coexistence of the ME signal with these resonances demonstrates that, although the reaction progressed, only partial conversion was achieved, with a substantial fraction of incompletely transesterified glycerides remaining.

This behavior is characteristic of heterogeneous catalytic systems and may be associated with diffusion limitations, an insufficient reaction time, or active site saturation. Higher temperatures favor the reaction by reducing medium viscosity and increasing molecular collision frequency, thereby accelerating transesterification kinetics.^32^

Gas chromatography (GC) results (Figure 7) corroborate the spectroscopic data, confirming that increasing the temperature and reaction time significantly enhances methyl ester yield, with the highest yields obtained at 200 °C for 120 min.

**Figure 7:**
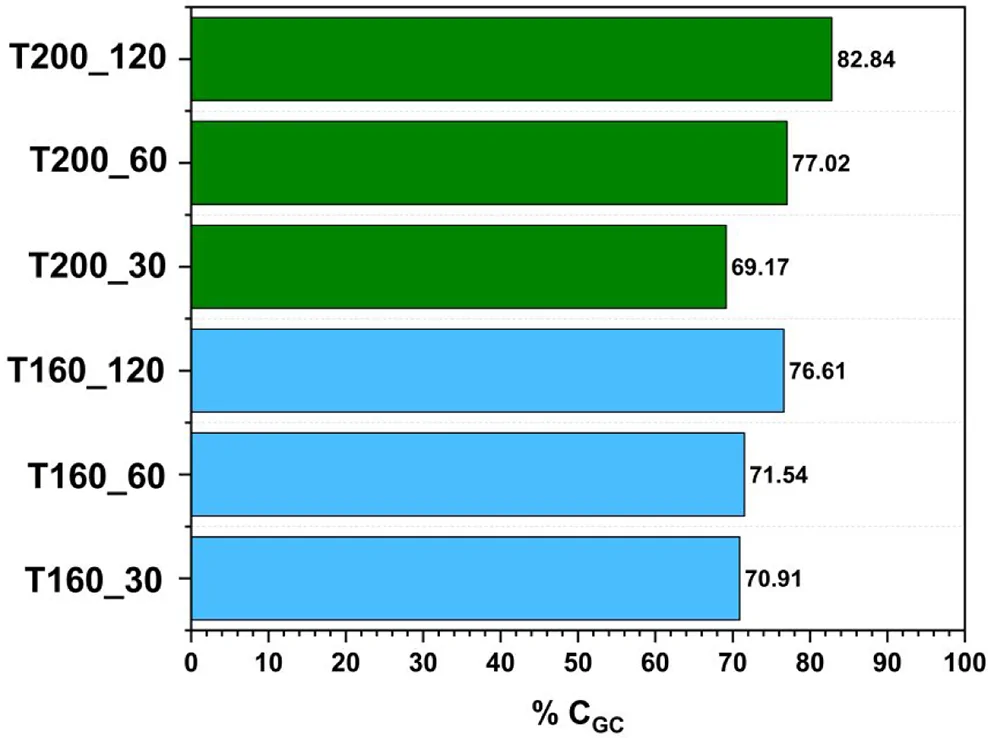
Methyl esters conversion (%) determined by gas chromatography after transesterification under different conditions.

Despite this similar behavior, very different conversion values were obtained by GC and ^1^H NMR analyses. Calculations using ^1^H NMR are based on the determination of the integrated areas of the peaks by deconvolution. Moreover, different equations can be used in the calculation. According to Jesus et al.,^35^ there are some drawbacks related to this method: the overlap of signals from converted and unconverted triglycerides; baseline correction, which may lead to mistakes in the integrated signal; and signal-to-noise ratio, which can make this discrepancy even larger. The errors may become even higher when intermediate compounds, such as monoglycerides and diglycerides, are present in the reaction product. On the other hand, GC calculations are based on standards that allow for more reliable calculations.

A comparison between catalysts indicates that transesterification activity depends not only on the number but also on the density of acid sites. The MoO_3_/ZnMoO_4_ catalyst (containing 68.64% of ZnMoO_4_) achieved methyl ester conversions of 76.61% at 160 °C and 82.84% at 200 °C after 120 min, both exceeding values reported for pure ZnMoO_4_ (16.18% and 57.85%) under identical conditions.^3^

The MoO_3_/ZnMoO_4_ composite shows lower efficiency compared to pristine α-MoO_3_, as α-MoO achieved 99.3% conversion after reaction at 150 °C for 120 min. Considering the percentage of ZnMoO_4_ and MoO_3_ in the present composite, conversions obtained at 150 and 160 °C for 120 min using the pristine materials were used to foresee the conversion obtained with the composite. According to this calculation, 42% conversion should be achieved by MoO_3_/ZnMoO_4_ at 160 °C/120 min, which is much smaller than the conversion of 76.61% obtained with the composite at this reaction condition. This behavior may be assigned to the interface effect observed in the FTIR and Raman spectra (Figure 3), which may be responsible for this synergic effect observed when both phases are present in the catalyst. This result is particularly significant given that ZnMoO_4_ contains approximately 55 times more acid sites than the composite. The superior performance of MoO_3_/ZnMoO_4_ may also be attributable to the greater accessibility of active sites, facilitated by its higher surface area (1.8 m^2^ g^–1^) relative to pristine ZnMoO_4_ (0.37 m^2^ g^–1^) and pristine α-MoO_3_ (0.50 m^2^ g^–1^), which may enhance the diffusion and adsorption of bulky molecules such as triacylglycerols.

Previous results from our research group^7,26^ indicate that active sites are related to the presence of Mo^5+^ reduced species in the material, associated with oxygen vacancies. We believe that these point defects also play an important role in molybdates, as a color change associated with Mo^6+^ reduction was also observed in the composites after catalytic tests.

#### 3.2.2 Esterification Reaction

The progressive reduction in acid value (Figures SI.4) reflects the consumption of free carboxylic groups,^36^ but also indicates that an additional esterification step would be required to bring the acid value below the 1 mg KOH g^–1^ threshold specified by EN 14214. All samples achieved conversions of 96–97% (Figure SI.4), indicating near-complete transformation of fatty acids into esters. Catalytic tests consistently outperformed blank reactions at 160 °C, which yielded conversions ranging from 29.3% at 30 min to 74.2% at 120 min.

Esterification conversion was also assessed by ^1^H NMR (Figure 8). The characteristic signal of the carboxylic acid (δ = 2.30–2.35 ppm) is substantially reduced after esterification of oleic acid, accompanied by the emergence of a peak at 3.6 ppm assigned to the −OCH_3_ group of methyl esters. Conversion ranged from 88 to 93.3%, with no systematic dependence on temperature or reaction time^36^ suggesting that milder conditions are sufficient for high esterification efficiency. Methyl ester formation was further confirmed by ^13^C NMR carbonyl signal analysis (Figure SI.5).

**Figure 8:**
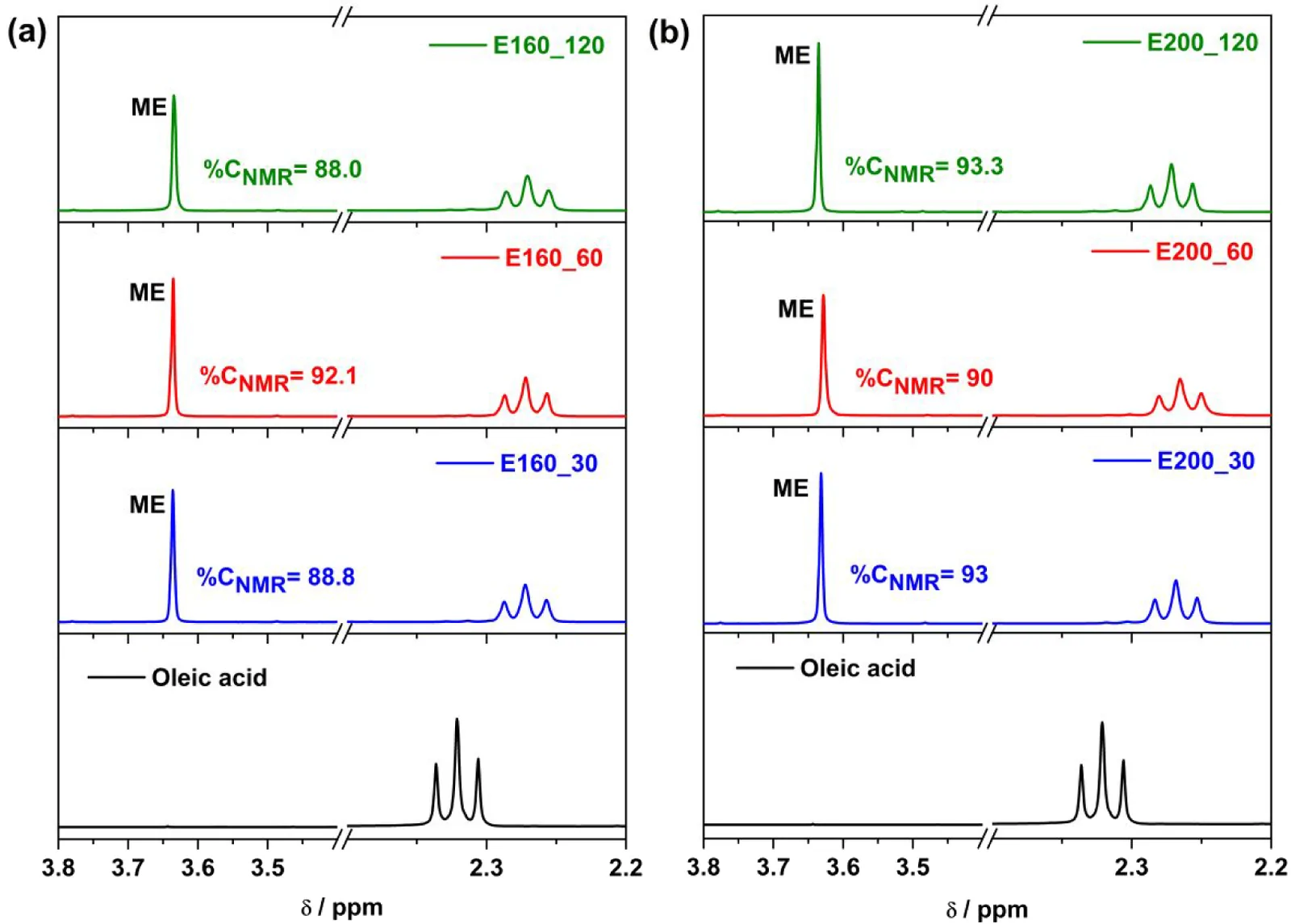
Expanded ^1^H NMR spectra (3.8–2.2 ppm) of oleic acid and esterification products at (a) 160 °C and (b) 200 °C.

The catalytic efficiency of MoO_3_/ZnMoO_4_ in esterification is attributed to its Lewis acid sites, which coordinate to the carbonyl oxygen of the carboxylic acid, increasing the electrophilicity of the carbonyl carbon and facilitating the nucleophilic attack by methanol, thereby promoting methyl ester formation.^37,38^

#### 3.2.3 Simultaneous Esterification-Transesterification Reaction (SET)

Based on the best results obtained from the individual reactions, SET reactions were conducted at 200 °C for 120 min using soybean oil acidified with 10% oleic acid (OA_10, A.I. = 22 mg KOH g^–1^), 20% oleic acid (OA_20, A.I. = 38 mg KOH g^–1^), and crude palm oil (CPO, A.I. = 13.9 mg KOH g^–1^). Products were characterized by the same techniques described above; results are summarized in Table 3.

**Table 3:** Methyl Esters Conversion (%) for SET Reactions at 200 °C for 120 min

Sample	Viscosity Reduction %	%C_A.I_	Acid Value mg KOH g^–1^	%C_CG_
**OA_10**	83.3	77	5.1	89.87
**OA_20**	84.9	94	2.3	88.85
**CPO**	85.0	91	1.2	96.22

The SET reactions effectively reduced both viscosity and acid value for all feedstocks. Acid values obtained for acidified soybean oil (Table 3) fall within the maximum limit established by the ANP Resolution n° 920/2023 for commercial biodiesel. Among the acidified samples, OA_10 showed the lowest FFA esterification conversion, while OA_20 displayed better overall performance. High conversion was also achieved with CPO, demonstrating the strong tolerance of MoO_3_/ZnMoO_4_ toward this naturally acidic feedstock, as further confirmed by GC data.

^1^H NMR analysis confirmed ME formation (Figure 9). The predominance of the methoxyl signal at 3.65 ppm, with %C_NMR_ exceeding 74% for all samples, demonstrates the positive effect of feedstock acidity compared to the pristine transesterification reaction (%C_NMR_ = 62.7%). In the ^13^C NMR spectra (Figure SI.6), signals at 62–70 ppm attributed to glycerol carbons persist in the reaction products,^34^ indicating incomplete conversion and suggesting that a second reaction step may be beneficial.

**Figure 9:**
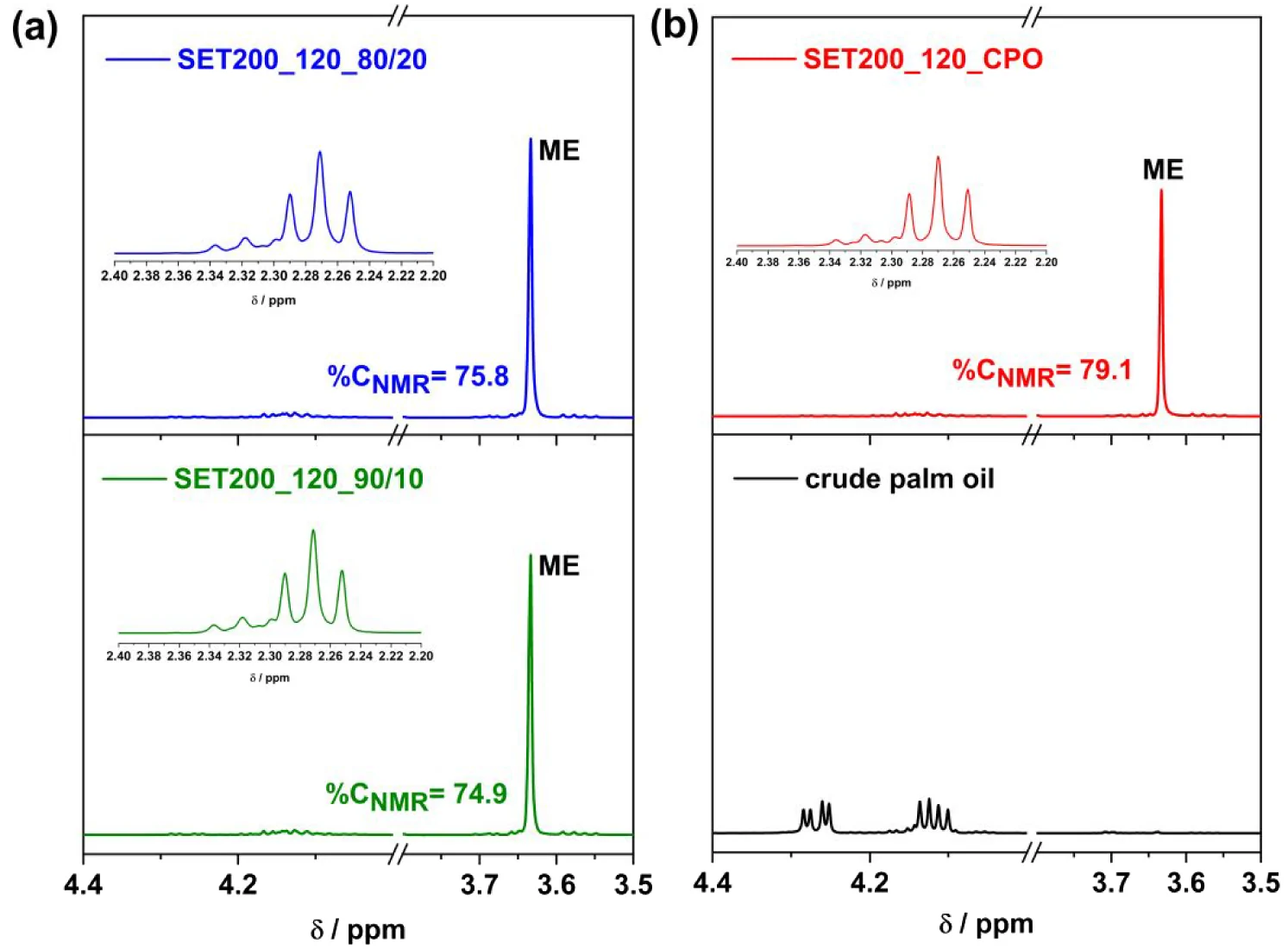
Expanded ^1^H NMR spectra (4.4–3.5 ppm) for SET reaction products using (a) acidified soybean oil and (b) crude palm oil.

GC results (Table 3) corroborate this trend, with conversions approaching 90% for acidified samples and reaching 96% for CPO, compared to 82.83% for the transesterification reaction alone. The SET200_120_CPO run achieved %CCG = 96.2% and %CIA = 91%, demonstrating a high catalytic efficiency. The presence of residual signs of carboxylic acid and glyceride signals nonetheless indicates incomplete conversion, further justifying a possible second reaction step.

A comparison with literature data is presented in Table SI.5. Despite the wide variation in reaction conditions across the literature, reported conversions for MoO_3_-based catalysts range from 85 to 99%, with the highest values obtained for pristine MoO_3_ at 200 °C using high methanol-to-oil ratios. For instance, Pinto et al.^12^ reported conversions of 93% and 95% for oil mixtures containing 10% and 20% oleic acid, respectively, using α-MoO_3_ at 150 °C for 4 h with a methanol:oil molar ratio of 45:1 and 0.5 wt % catalyst loading. In the present work, MoO_3_/ZnMoO_4_ achieved conversions of approximately 90% for the same oleic acid contents under milder conditions—a methanol:oil molar ratio of 12:1 and a reaction time of only 2 h. Wang, Xie, and Guo^1^ developed an acidic MoO_3_/ZrO_2_/KIT-6 catalyst bearing both Brønsted–Lowry and Lewis acid sites, achieving 92.7% conversion for oil containing 20% oleic acid; however, these results required 12 h of reaction, a methanol:oil molar ratio of 20:1, and a substantially higher catalyst loading of 12%.

A more direct comparison can be drawn with Colaço et al.,^3^ who employed ZnMoO_4_ as a catalyst for SET reactions using soybean oil containing 20% oleic acid, obtaining 69.5% methyl ester conversion at 160 °C for 120 min. For pristine MoO_3_, 85.2% conversion was obtained at 150 °C for 120 min.^26^ Under identical reaction conditions, the MoO_3_/ZnMoO_4_ composite reached 86.7% conversion at 160 °C for 120 min, a higher value than either constituent phase alone, particularly noteworthy given the composite composition (68.64% ZnMoO_4_, 31.35% MoO_3_). This result provides strong evidence for a synergistic effect, likely arising from interface interactions as indicated by FTIR and Raman spectroscopy.

Although ZnMoO_4_ presents a higher Lewis acid site density (181 μmol g^–1^) than the composite (3.30 μmol g^–1^), the superior catalytic performance of MoO_3_/ZnMoO_4_ may be attributed to the emergence of new Lewis acid sites associated with the presence of MoO_3_ and interface effects, as well as to the higher surface area of the composite (1.8 m^2^ g^–1^ vs 0.37 m^2^ g^–1^), which likely enhances active site accessibility. Compared to pristine MoO_3_ at 200 °C for 2 h, the composite shows slightly lower conversions for acidified soybean oil (∼90% vs 94–95%), consistent with the trend in Table SI.5. The disadvantage of pristine MoO_3_ is its strong tendency toward preferential orientation, which reduces accessible active sites, as previously reported by our research group.^7^

The MoO_3_/ZnMoO_4_ composite was applied in a reuse test, using CPO as raw material. After each reuse, the catalyst was recovered, washed with *n*-hexane to remove adsorbed organic species, and dried at 150 °C for 2 h before being reused. Results of conversion obtained by gas chromatography and acid value variation are displayed in Figure 10. Conversion of the triglycerides into methyl esters was constant after three consecutive reuse tests, while a small decrease in the conversion of the free fatty acids was observed. This decrease was more pronounced in the first reuse test, with a drop of 8%, whereas less than a 5% decrease was observed in the third reuse test. This result confirms the stability of the catalyst and emphasizes its possible use as a catalyst for biodiesel production using acid feedstocks.

**Figure 10:**
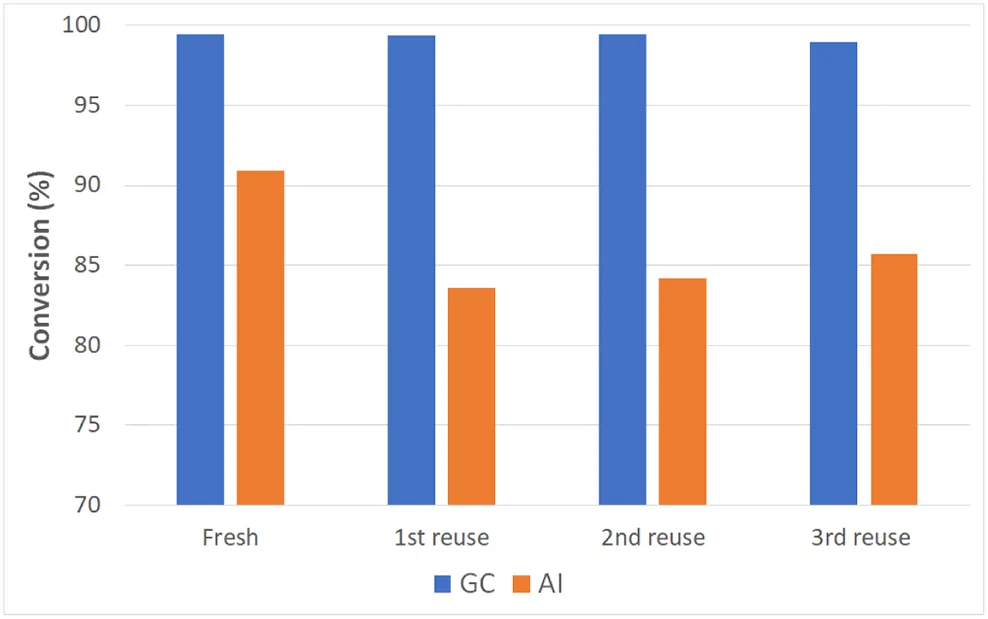
Conversion of CPO into methyl esters after three consecutive tests of reuse using MoO_3_/ZnMoO_4_ as catalyst.

## 4 Conclusion

The MoO_3_/ZnMoO_4_ composite was successfully synthesized via the modified Pechini method, in a one-pot reaction, yielding a two-phase material with a predominance of the ZnMoO_4_ phase. Pyridine-probe FTIR analysis revealed the emergence of new acid sites absent in pure ZnMoO_4_, indicating that interface effects between the two phases modify the surface acid properties of the composite, as corroborated by both IR and Raman spectroscopy. In transesterification, the composite achieved methyl ester conversions of 76.61% at 160 °C and 82.84% at 200 °C after 120 min, substantially exceeding values reported for pure ZnMoO_4_ (16.18% and 57.85%, respectively), under identical conditions. In SET reactions, conversion increased further, even when using crude palm oil as feedstock, with 91% FFA conversion based on acid index and 96.22% methyl ester yield by gas chromatography. Reuse tests using crude palm oil demonstrated that the MoO_3_/ZnMoO_4_ composite maintained high catalytic stability throughout three consecutive reuses. These results demonstrate that the MoO_3_/ZnMoO_4_ composite exhibits enhanced transesterification activity and greater tolerance toward high-acidity feedstocks, likely due to the generation of new Lewis acid sites and improved active site accessibility arising from the increased surface area.

## Supplementary Material



## Notes

Characterization of the catalyst: XRD pattern after Rietveld analysis; assignments of IR bands; assignments of Raman peaks; zeta potential data; characterization of the products after transesterification reaction: viscosity data and acid values; ^13^C NMR spectra; characterization of the products after esterification reaction: viscosity data; conversion percentages according to the acid value; ^13^C NMR spectra; characterization of the products after SET reaction: ^13^C NMR spectra; literature data (PDF)
