# Supplementary material for: MoO3/ZnMoO4 Composite Obtained in a One-Pot Reaction and Applied in the Production of Biodiesel from Acid Feedstocks
Source: ACS Omega. 2026 Sep 22;11(39):59251–61. doi: 10.1021/acsomega.6c05074 (PMC13647835; doi:10.1021/acsomega.6c05074)
Supplement: Supplementary file 1 [file ao6c05074_si_001.pdf]

$MoO_3/ZnMoO_4$ composite obtained in one-pot reaction and applied in the production of biodiesel from acid feedstocks

Heritier Kabamba Subissay[1], Amanda Soares de Sousa[1], Ana Cristina F. M. Costa[2], Ieda Maria Garcia dos Santos[1*]

[1] Núcleo de Pesquisa e Extensão – Laboratório de Combustíveis e Materiais, Universidade Federal da Paraíba, Joao Pessoa - PB, CEP 58051-900, Brazil

[2]Laboratório de Síntese dos Materiais Cerâmicos (LabSMaC), Universidade Federal de Campina Grande, 58429-900 Campina Grande-PB, Brazil

*Corresponding author: ieda@quimica.ufpb.br (I.M.G.S)

ACS Omega

Results and discussion

Characterization of the $MoO_3/ZnMoO_4$ composite

Rietveld analysis was performed, with an excellent fit, as demonstrated in Figure SI.1.

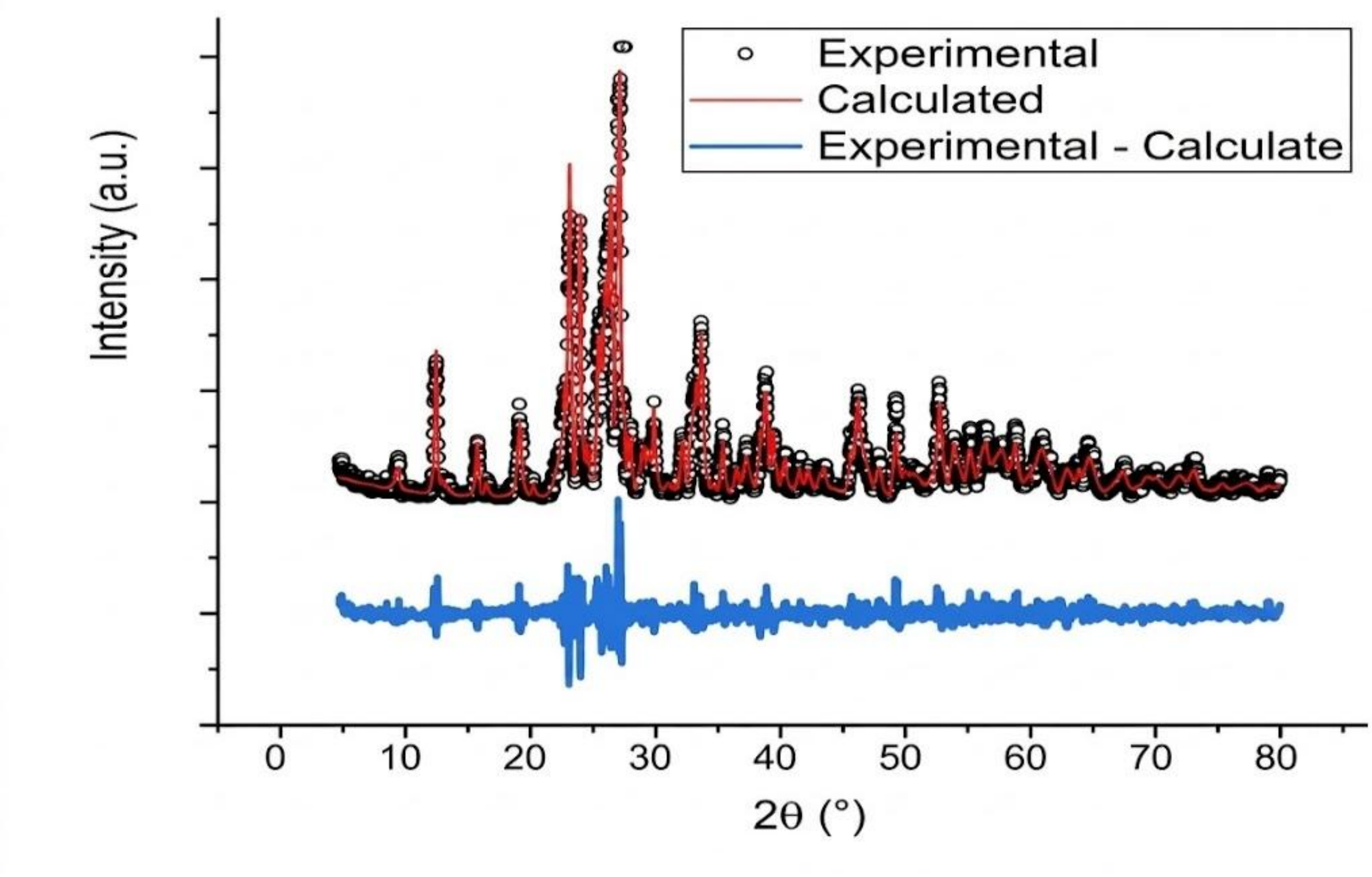

***Figure SI.1**. Comparison between experimental data and theoretical (calculated) data for the* $MoO_3/ZnMoO_4$ *phases, in agreement to the Rietveld method*

Spectroscopic characterization of the composite was performed to bring a better understanding of the interface between the two phases. Tables SI.1 and SI.2 display the assignments of the IR spectrum and of the Raman spectrum, respectively.

Table SI.1. *Assignments of the IR bands for the $MoO_3/ZnMoO_4$ composite*
ν: stretching; **δ**: bending; **sci**: scissor; **as**: asymmetric; **s**: symmetric

REGION (cm^{-1})		ASSIGNMENT	PHASE	REFERENCE
**Composite**	**Pristine materials [3,7]**			
433	434	δ (MoO_4), δ (ZnO_6)	$ZnMoO_4$	Silva Filho *et al.*[1]
454	-	sci (MoO_4), δ (ZnO_6)	$ZnMoO_4$	Silva Filho *et al.*[1]
514	486	δ (O-Mo-O)	MoO_3	Medeiros, Farias, and Santos[2]
566	595	Mo(3)-O	MoO_3	Medeiros, Farias, and Santos[2]
756, 803	753, 800	ν_{as} (MoO_4), δ (ZnO_6)	$ZnMoO_4$	Silva Filho *et al.*[1]
845, 868	868	ν_{as} (MoO_4), ν_{as} (ZnO_6)	$ZnMoO_4$	Silva Filho *et al.*[1]
892	892	ν_s (MoO_4), ν_{as} (ZnO_6)	$ZnMoO_4$	Silva Filho *et al.*[1]
910	910	ν_s (MoO_4), ν_s (ZnO_6)	$ZnMoO_4$	Silva Filho *et al.*[1]
934, 951, 956	953	ν_s (MoO_4), ν_{as} (ZnO_6)	$ZnMoO_4$	Silva Filho *et al.*[1]
986	997	ν (Mo=O) terminal	MoO_3	Medeiros, Farias, and Santos[2]

Table SI.2. *Assignments of the Raman peaks for the $MoO_3/ZnMoO_4$ composite*
ν: stretching; **δ**: bending; **sci**: scissor; **T**: translational; **as**: asymmetric; **s**: symmetric

REGION (cm^{-1})		ASSIGNMENTS	PHASE	REFERENCE
**Composite**	**Pristine materials [6,7]**			
116	116	T (rede)	MoO_3	Medeiros, Farias, and Santos[2]
130	128	T (rede)	MoO_3	Medeiros, Farias, and Santos[2]
157	157	T (rede)	MoO_3	Medeiros, Farias, and Santos[2]
199	195	τ (Mo=O=Mo)	MoO_3	Medeiros, Farias, and Santos[2]
217	216	δ (Mo(2)-O)	MoO_3	Medeiros, Farias, and Santos[2]
246	244	τ (Mo=O=Mo)	MoO_3	Medeiros, Farias, and Santos[2]
283	283	δ (Mo=O=Mo)	MoO_3	Medeiros, Farias, and Santos[2]
292	290	δ (Mo=O=Mo)	MoO_3	Medeiros, Farias, and Santos[2]
338	336	δ (Mo-O-Mo)	MoO_3	Medeiros, Farias, and Santos[2]
363	366	δ (Mo-O-Mo)	MoO_3	Medeiros, Farias, and Santos[2]
382	379	δ (Mo-O-Mo)	MoO_3	Medeiros, Farias, and Santos[2]
473	470	ν_{as} (Mo(3)-O)	MoO_3	Medeiros, Farias, and Santos[2]
666	667	ν_{as} (Mo-O)	MoO_3	Medeiros, Farias, and Santos[2]
817	818	ν_s (Mo-O-Mo)	MoO_3	Medeiros, Farias, and Santos[2]
860	830	ν_{as} (MoO_4), δ (ZnO_6)	$ZnMoO_4$	Silva Filho *et al.*[1]
884	850	ν_{as} (MoO_4), δ (ZnO_6)	$ZnMoO_4$	Silva Filho *et al.*[1]
891	871	ν_s (MoO_4), δ (ZnO_6)	$ZnMoO_4$	Silva Filho *et al.*[1]
935	920	ν_s (MoO_4), δ (ZnO_6)	$ZnMoO_4$	Silva Filho *et al.*[1]
943	935	ν_s (MoO_4), ν_{as} (ZnO_6)	$ZnMoO_4$	Silva Filho *et al.*[1]
954	945	ν_s (MoO_4), ν_{as} T(ZnO_6)	$ZnMoO_4$	Silva Filho *et al.*[1]
966	957	ν_s (MoO_4), ν_s (ZnO_6)	$ZnMoO_4$	Silva Filho *et al.*[1]
995	994	ν (Mo=O) terminal	MoO_3	Medeiros, Farias, and Santos[2]

Zeta potential is a fundamental parameter for determining the electrical charge present on the surface of a material, directly influencing its catalytic behavior. This measurement was performed to evaluate the surface charge density of the $MoO_3/ZnMoO_4$ catalyst at different pH values and to determine the point of zero charge (PZC) in aqueous media. For this purpose, a suspension containing $NaNO_3$ (0.1 mmol L^{-1}) as supporting electrolyte and the catalyst at a concentration of 0.1 g L^{-1} was prepared. 10 mL aliquots

of the suspension were kept under constant stirring at room temperature for 24 h. The pH of the suspensions was adjusted to 2, 4, 6, 8, 10, and 12 using solutions of HNO_3 and NaOH (0.1 mol L^{-1}). The analyses were performed in duplicate. After pH stabilization, the zeta potential was determined using a Zetasizer Nano Series analyzer at room temperature, allowing the characterization of the surface charge and colloidal stability of the system as a function of pH.

In the present study, experimental analysis revealed that $MoO_3/ZnMoO_4$ composite exhibits predominantly negative surface across the entire pH range analyzed, reaching a maximum of -24.9 mV at pH 12, such that it was not possible to determine the point of zero charge (PZC), as shown in Figure SI.2. This behavior is consistent with previous studies that report similar characteristics for molybdenum oxide (MoO_3) and zinc molybdate ($ZnMoO_4$), highlighting their strong tendency to deprotonation in aqueous media. [3,4]

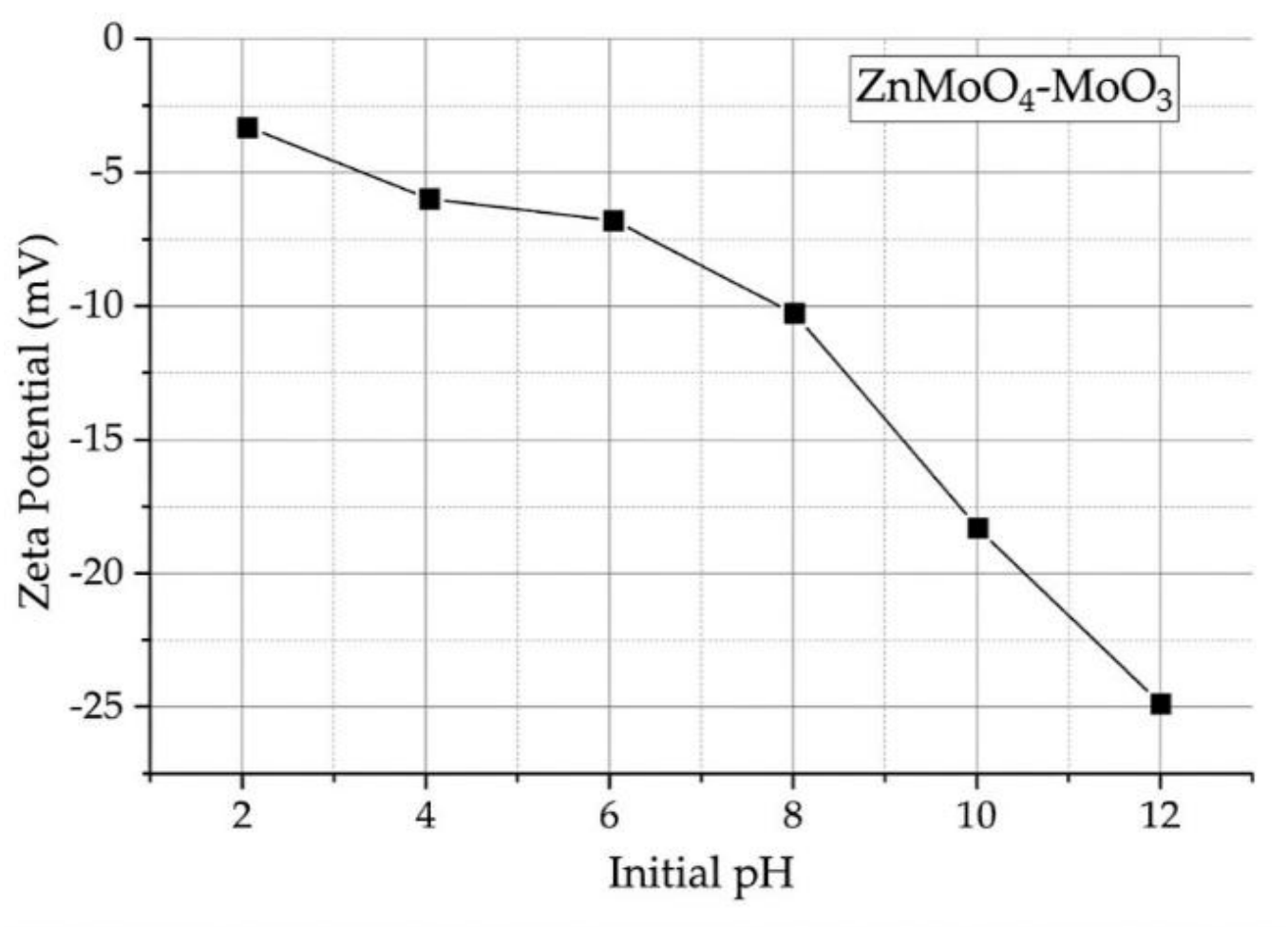

Figure SI.2. *Zeta potential variation as a function of pH values of the* $MoO_3/ZnMoO_4$ *composite*

Catalytic tests

Transesterification reaction

The viscosity results indicated that the $MoO_3/ZnMoO_4$ system leads to a product which agrees with the ANP regulatory limits (3.0–5.0 $mm^2\ s^{-1}$), as displayed in Table SI.3, demonstrating its catalytic efficiency. [4,5]

Table SI.3: *Viscosity data and acid values after transesterification reactions*

**Catalytic tests**	**Viscosity ($mm^2 s^{-1}$)**	**Reduction in viscosity (%)**	**Acid value (mg KOH/g)**
Soybean oil	34.79	0	0.5
T200_30	9.9	70.9	0.09
T200_60	5.61	83.9	0.18
T200_120	4.62	86.7	0.40
T160_30	8.68	75.0	0.47
T160_60	4.06	88.3	0.12
T160_120	4.62	86.7	0.34

As illustrated in Table SI.3, the kinematic viscosity progressively decreased with increasing reaction time, both at 200 °C and 160 °C. At 200 °C, the values decreased from 9.90 $mm^2 s^{-1}$ (T200_30) to 5.61 $mm^2 s^{-1}$ (T200_60) and 4.62 $mm^2 s^{-1}$ (T200_120), while at 160 °C, T160_30 showed 8.68 $mm^2 s^{-1}$, and T160_60 and T160_120 reached 4.06 and 4.62 $mm^2 s^{-1}$, respectively, demonstrating that the increase in reaction time compensates for the lower temperature.

The greatest viscosity reductions occurred at T160_60 (88.3%), T160_120 (86.7%), and T200_120 (83.7%), highlighting T160_60 as the most efficient condition and in compliance with ANP limits (2.0–4.5 $mm^2 s^{-1}$ at 40 °C). The acid value (A.I.) remained below 0.50 mg KOH g^{-1} in all samples, in accordance with ANP Resolution No. 45/2014. A random behavior was observed as a function of time and temperature, but A.I. values were always lower than that of soybean oil, due to the consumption of free fatty acids (FFAs) due to an esterification reaction. [6,7]

The reaction products were also characterized by ^{13}C NMR, as displayed in Figure SI.3.

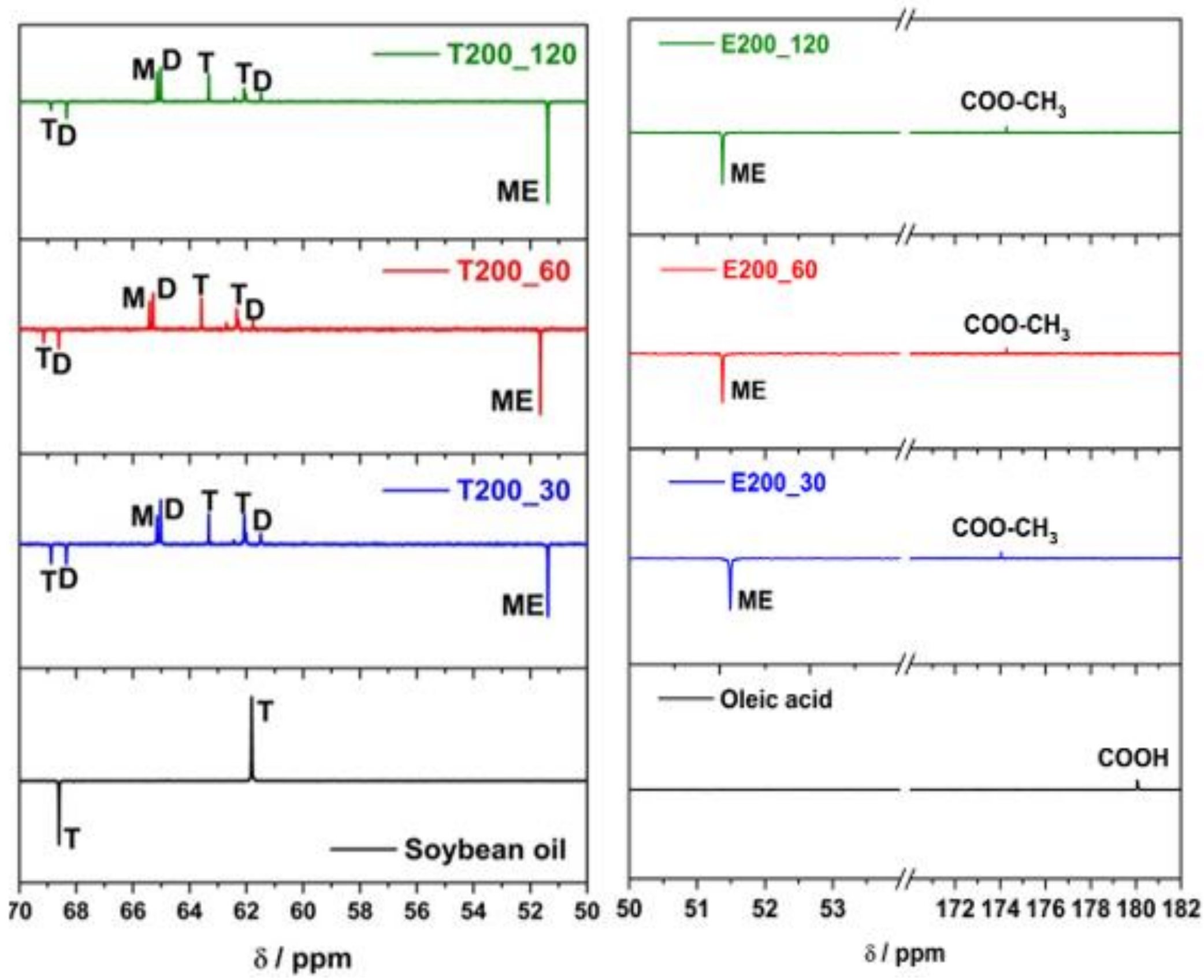

(a) (b)

Figure SI.3 *^{13}C NMR spectra of the raw materials and of the products obtained after catalytic reaction at 200 °C. (a) transesterification reaction, with the characteristic peak of the methoxyl group of the methyl ester (ME) and residual signals attributed to triglycerides (T), diglycerides (D) and monoglycerides (M); (b) esterification reaction*

The characteristic signal of the carboxylic group (δ 180–182 ppm) is intense in pure oleic acid and shows a significant reduction after esterification. At 160 °C, this peak is still detectable, indicating partial conversion, while at 200 °C it practically disappears, confirming greater consumption of oleic acid. This behavior corroborates the positive effect of temperature in the esterification reaction, as reported by previous studies[8,9]. In the δ 173–174 ppm region, signals attributed to the carbonyl carbon of the ester groups ($COO–CH_3$) are observed at 200 °C, indicating higher conversion, while at 160 °C they show lower intensity, consistent with intermediate conversion. The presence of these signals confirms the formation of esters.[10]

Esterification reaction

The viscosity values ranged from 4.50 to 5.18 $mm^2\ s^{-1}$ are presented in Table SI.4. Almost all values are below the upper limit established by EN 14214 and National Agency of Petroleum, Natural Gas, and Biofuels (ANP) regulations, which stipulates a range of 3.0 to 5.0 $mm^2\ s^{-1}$ for biodiesel.[5]

***Table SI.4**: Viscosity values after esterification reactions*

**Catalytic Test**	**Viscosity ($mm^2\ s^{-1}$)**	**Reduction in Viscosity (%)**
Oleic acid	34.79	-
T160_30	5.18	73.0
T160_60	4.81	75.0
T160_120	4.75	75.3
T200_30	4.65	76.6
T200_60	4.62	76.0
T200_120	4.58	75.8

The results indicate a significant reduction in the kinematic viscosity of oleic acid after the catalytic reactions, evidencing its conversion into methyl esters with lower molecular weight and greater fluidity. At 160 °C, the viscosity was reduced to 5.18 $mm^2\ s^{-1}$ in 30 min (73.0%) and 4.75 $mm^2\ s^{-1}$ in 120 min (75.3%), stabilizing after 60 minutes, suggesting that most of the conversion occurs in the initial steps. At 200 °C, the values were even lower, reaching 4.65 $mm^2\ s^{-1}$ in 30 min (76.6%), remaining practically constant until 120 minutes, indicating the approach of reaction equilibrium. These results confirm that increasing the temperature favors the kinetics of esterification, accelerating the conversion of free fatty acids into methyl esters.[11]

Reduction in the acid value and the conversion values are displayed in Figure SI.4 and indicate that a high conversion occurred, even at low temperatures.

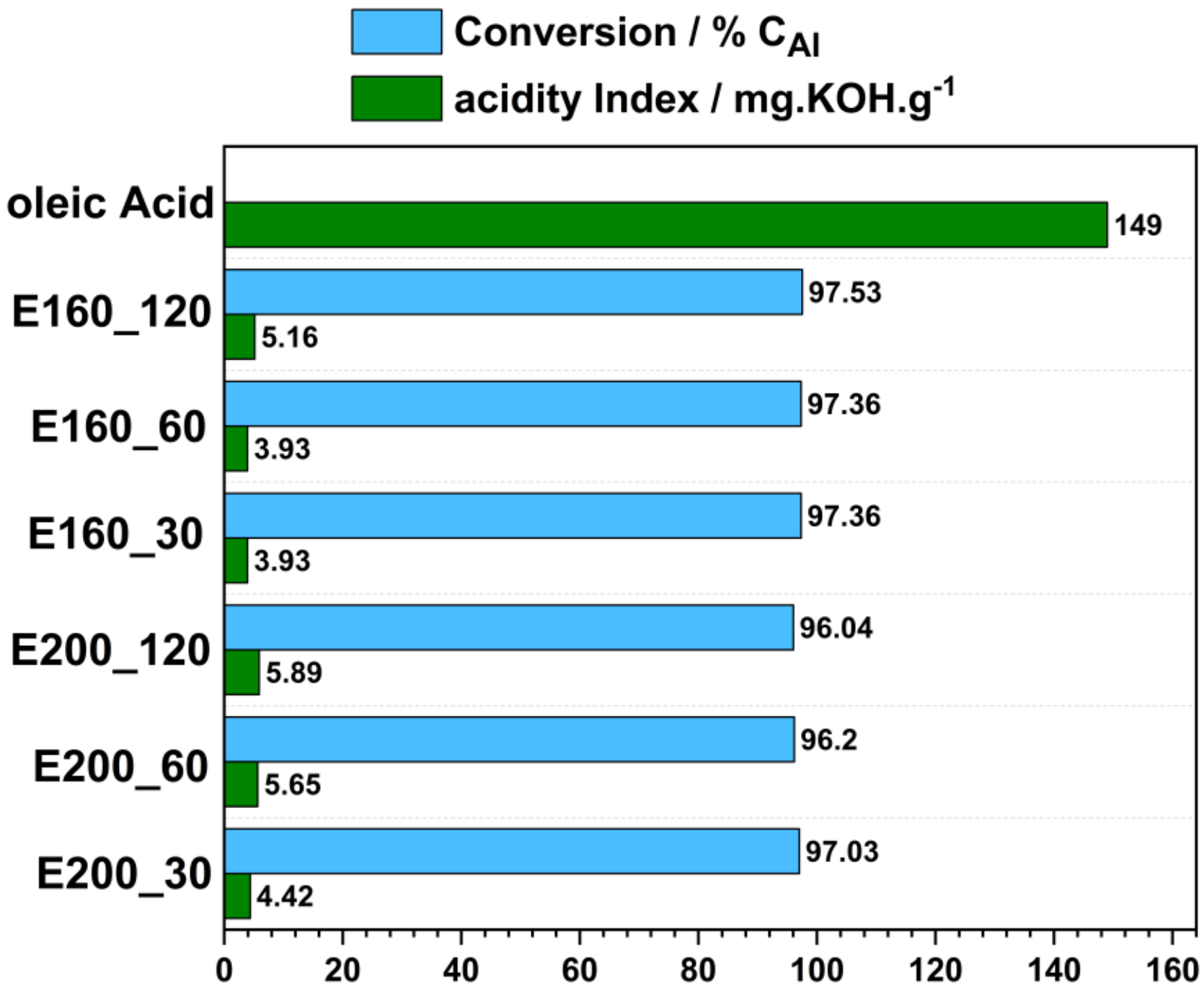

Figure SI.4. *Results of acid value and the respective conversion after catalytic test at 160 and 200 °C.*

The reaction products were also characterized by ^{13}C NMR, as displayed in Figure SI.5. The characteristic signal of the carboxylic group (δ 180–182 ppm) is intense in pure oleic acid and shows a significant reduction after esterification. At 160 °C, this peak is still detectable, indicating partial conversion, while at 200 °C it practically disappears, confirming greater consumption of oleic acid. This behavior corroborates the positive effect of temperature in the esterification reaction, as reported by previous studies[8,9]. In the δ 173–174 ppm region, signals attributed to the carbonyl carbon of the ester groups (COO–CH_3) are observed at 200 °C, indicating higher conversion, while at 160 °C they show lower intensity, consistent with intermediate conversion. The presence of these signals confirms the formation of esters.[10]

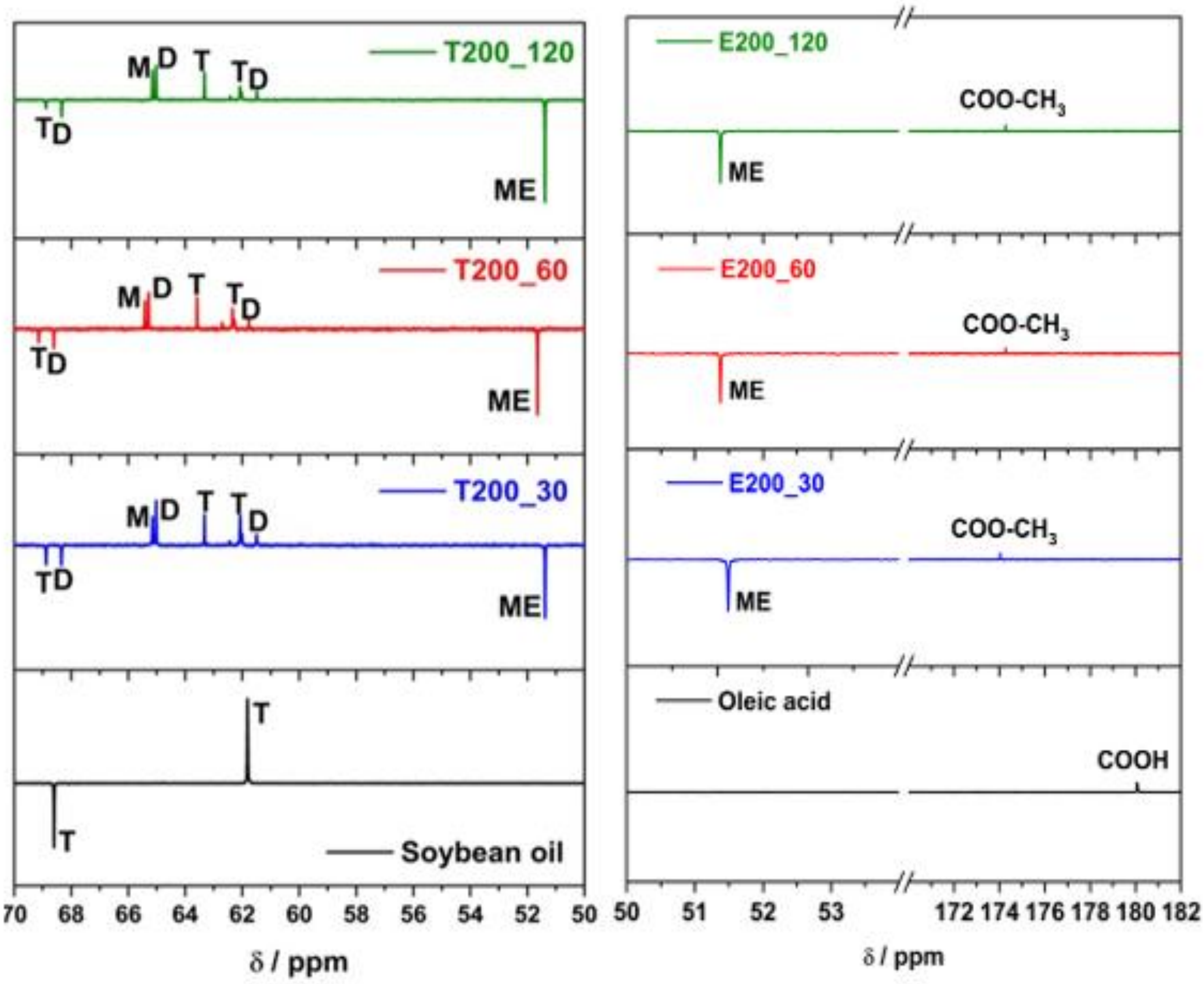

Figure SI.5 *^{13}C NMR spectra of the raw materials and of the products obtained after catalytic reaction at 200 °C. (a) transesterification reaction, with the characteristic peak of the methoxyl group of the methyl ester (ME) and residual signals attributed to triglycerides (T), diglycerides (D) and monoglycerides (M); (b) esterification reaction*

The reaction products were also characterized by ^{13}C NMR, as displayed in Figure SI.6.

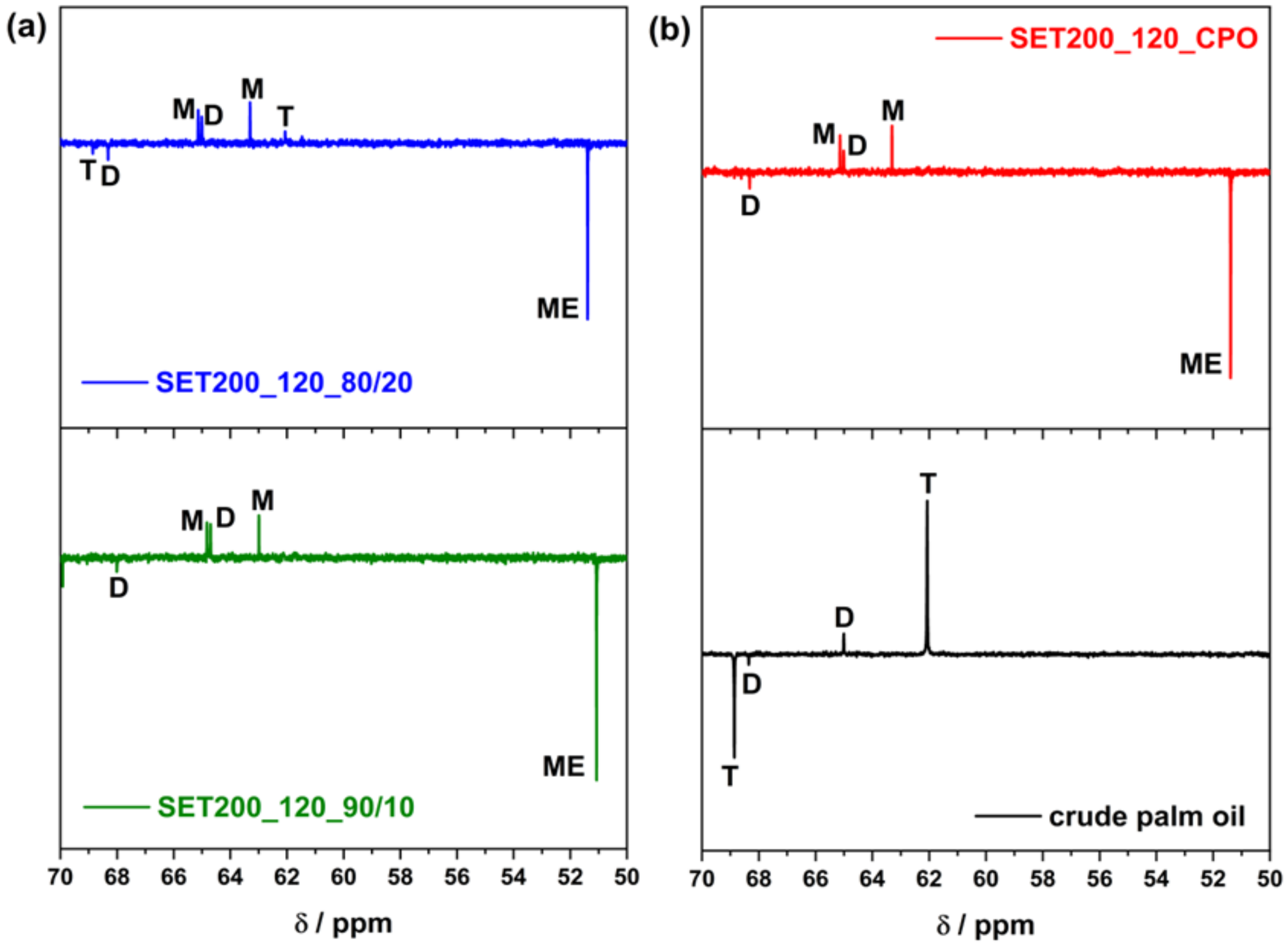

Figure SI.6. *Magnifications of the ^{13}C NMR spectra (70-50 ppm) after SET reactions for the samples using (a) acidified oil (b) crude palm oil.*

Table SI.5 displays data for SET reactions performed with $ZnMoO_4$, MoO_3 and composites containing MoO_3, for comparison with the results obtained in the present work.

Table SI.5. Literature data displaying results of SET reactions using pristine $ZnMoO_4$, pristine MoO_3 and MoO_3 composites, as catalysts.

**Reference**	**Catalyst**	**Raw Materials**	**Alcohol:Oil Ratio**	**Temperature / Time**	**Conversion (%)**
Colaço *et al.*[12]	$ZnMoO_4$	Acidified Soybean Oil	12:1	160 °C / 2 h<br>160 °C / 4 h	69.5%<br>73.0%
Gonçalves *et al.*[13]	$MoO_3/SrFe_2O_4$	Waste Soybean Oil	40:1	160 °C / 4 h	95.4%
Silva *et al.*[14]	α-MoO_3	Residual Oil	15:1 / 20:1	160°C / 200°C	93% / 99%
Silva *et al.*[15]	MoO_3/Graphene	Waste Soybean Oil	32:1	140 °C / 5h	95.6%
Brito *et al.*[16]	MoO_3/Nb_2O_5	Residual Oil	20:1	145 °C / 2.5 h	94.2%
Wang, Xie and Guo[17]	$MoO_3/ZrO_2/KIT_6$	20% acidified oil	20:1	130 °C / 12 h	92.7%
Navajas *et al.*[18]	MoO_3-Al_2O_3	Acidified sunflower oil	12:1	100 °C / 1 h	85%
Alhassan; Rashid and Taufiq-Yap [19]	Fe-Mn-MoO_3/ZrO_2	Waste Cooking Oil	25:1	200 °C / 5 h	95.6%
Pinto *et al.*[20]	α-MoO_3	Acidified oil (10%/20%)	45:1	150 °C / 4 h	93% / 95%

References

(1) da Silva Filho, J. G.; Saraiva, G. D.; de Castro, A. J. R.; Neto, V. O. S.; Saraiva-Souza, A.; Silva, C. B.; Lima, J. A.; Teixeira, A. M. R.; Freire, P. T. C.; Paraguassu, W.; de Sousa, F. F. Vibrational Spectroscopy Study and Ab Initio Calculation on $ZnMoO_4$ System. *J. Mol. Struct.* **2020**, *1206*, 127776. https://doi.org/10.1016/J.MOLSTRUC.2020.127776.

(2) Medeiros, S. A. S. L. de; Farias, A. F. F.; Santos, I. M. G. dos. Synthesis of Molybdenum Trioxide with Different Structures and Microstructures by the Modified Pechini Method: A New Methodological Approach. *Rev. Virtual Quim.* **2021**, *13* (2), 494–508. https://doi.org/10.21577/1984-6835.20200157.

(3) Kumar, P.; Verma, S.; Korošin, N. Č.; Žener, B.; Štangar, U. L. Increasing the Photocatalytic Efficiency of $ZnWO_4$ by Synthesizing a Bi2WO6/ZnWO4 Composite Photocatalyst. *Catal. Today* **2022**, *397–399*, 278–285. https://doi.org/10.1016/J.CATTOD.2021.09.012.

(4) Meher, L. C.; Vidya Sagar, D.; Naik, S. N. Technical Aspects of Biodiesel Production by Transesterification—a Review. *Renew. Sustain. Energy Rev.* **2006**, *10* (3), 248–268. https://doi.org/10.1016/J.RSER.2004.09.002.

(5) *Agência Nacional Do Petróleo, Gás Natural e Biocombustíveis. Resolução ANP Nº 920, de 4 de Abril de 2023: Estabelece a Especificação Do Biodiesel e as Obrigações Quanto Ao Controle Da Qualidade*; Brasília, 2023. https://atosoficiais.com.br/anp/resolucao-n-920-2023-estabelece-a-especificacao-do-biodiesel-e-as-obrigacoes-quanto-ao-controle-da-qualidade-a-serem-atendidas-pelos-agentes-economicos-que-comercializem-o-produto-em-territorio-nacional?origin=instituicao.

(6) Nunes, R. F.; Paula, D. C. G. de; Martins, M. G. Produção de Biodiesel Do Óleo

de Amendoim Por Transesterificação Metílica e Aplicação Da Superfície de Resposta. *Sci. Plena* **2021**, *17* (4), 2021. https://doi.org/10.14808/SCI.PLENA.2021.044201.
(7) Sharma, Y. C.; Singh, B.; Upadhyay, S. N. Advancements in Development and Characterization of Biodiesel: A Review. *Fuel* **2008**, *87* (12), 2355–2373. https://doi.org/10.1016/J.FUEL.2008.01.014.
(8) Marchetti, J. M.; Errazu, A. F. Esterification of Free Fatty Acids Using Sulfuric Acid as Catalyst in the Presence of Triglycerides. *Biomass and Bioenergy* **2008**, *32* (9), 892–895. https://doi.org/10.1016/J.BIOMBIOE.2008.01.001.
(9) Knothe, G.; Gerpen, J. V; KRAHL, J.; RAMOS, L. P. Manual de Biodiesel, 1ª Edição. *São Paulo Edgard Blucher* **2006**, 133.
(10) Schuchardt, U.; Sercheli, R.; Matheus, R. Transesterification of Vegetable Oils : A Review General Aspects of Transesterification Transesterification of Vegetable Oils Acid-Catalyzed Processes Base-Catalyzed Processes. *Journal* **1998**, *9* (1), 199–210.
(11) Freedman, B.; Butterfield, R. O.; Pryde, E. H. Transesterification Kinetics of Soybean Oil 1. *J. Am. Oil Chem. Soc. 1986 6310* **1986**, *63* (10), 1375–1380. https://doi.org/10.1007/BF02679606.
(12) Colaço, L. A.; Sousa, A. S.; C. F. M. Costa, A.; F. F. Farias, A.; M. G. Santos, I. Zinc Molybdate: A New Catalyst for Biodiesel Synthesis by Simultaneous Esterification/Transesterification Reaction. *Fuel* **2024**, *371*, 132066. https://doi.org/10.1016/j.fuel.2024.132066.
(13) Arrais Gonçalves, M.; Karine Lourenço Mares, E.; Roberto Zamian, J.; Narciso da Rocha Filho, G.; Rafael Vieira da Conceição, L. Statistical Optimization of Biodiesel Production from Waste Cooking Oil Using Magnetic Acid Heterogeneous Catalyst MoO3/SrFe2O4. *Fuel* **2021**, *304*, 121463. https://doi.org/10.1016/j.fuel.2021.121463.
(14) Silva, A. L. da; Pereira, H. de L.; Sales, H. B.; Dionízio, J. K.; Alves, M. C. F.; Guedes, D. G.; Luna, C. B. B.; Costa, A. C. F. de M. Optimization of Biodiesel Production Process Using MoO3 Catalysts and Residual Oil: A Comprehensive Experimental 23 Study. *Molecules* **2024**, *29* (10), 2404. https://doi.org/10.3390/molecules29102404.
(15) da Silva, P. M. M.; Gonçalves, M. A.; da Luz Corrêa, A. P.; da Luz, P. T. S.; Zamian, J. R.; da Rocha Filho, G. N.; da Conceição, L. R. V. Preparation and Characterization of a Novel Efficient Catalyst Based on Molybdenum Oxide Supported over Graphene Oxide for Biodiesel Synthesis. *Renew. Energy* **2023**, *211*, 126–139. https://doi.org/10.1016/J.RENENE.2023.04.131.
(16) de Brito, V. L.; Gonçalves, M. A.; dos Santos, H. C. L.; da Rocha Filho, G. N.; da Conceição, L. R. V. Biodiesel Production from Waste Frying Oil Using Molybdenum over Niobia as Heterogeneous Acid Catalyst: Process Optimization and Kinetics Study. *Renew. Energy* **2023**, *215*, 118947. https://doi.org/10.1016/J.RENENE.2023.118947.
(17) Wang, Q.; Xie, W.; Guo, L. Molybdenum and Zirconium Oxides Supported on KIT-6 Silica : A Recyclable Composite Catalyst for One e Pot Biodiesel Production from Simulated Low-Quality Oils. *Renew. Energy* **2022**, *187*, 907–922. https://doi.org/10.1016/j.renene.2022.01.122.
(18) Navajas, A.; Reyero, I.; Jiménez-Barrera, E.; Romero-Sarria, F.; Llorca, J.; Gandía, L. M. Catalytic Performance of Bulk and Al_2O_3-Supported Molybdenum Oxide for the Production of Biodiesel from Oil with High Free Fatty Acids Content. *Catalysts* **2020**, *10*, 1–14.

(19) Alhassan, F. H.; Rashid, U.; Taufiq-Yap, Y. H. Synthesis of Waste Cooking Oil Based Biodiesel via Ferric-Manganese Promoted Molybdenum Oxide / Zirconia Nanoparticle Solid Acid Catalyst: Influence of Ferric and Manganese Dopants. *J. Oleo Sci.* **2015**, *64* (5), 505–514. https://doi.org/10.5650/jos.ess14228.
(20) Pinto, B. F.; Garcia, M. A. S.; Costa, J. C. S.; de Moura, C. V. R.; de Abreu, W. C.; de Moura, E. M. Effect of Calcination Temperature on the Application of Molybdenum Trioxide Acid Catalyst: Screening of Substrates for Biodiesel Production. *Fuel* **2019**, *239*, 290–296. https://doi.org/10.1016/j.fuel.2018.11.025.
